# Synthetic preimplantation factor ameliorates the severity of radiotherapy-induced heart disease in a rat model

**DOI:** 10.3389/fcvm.2026.1946843

**Published:** 2026-10-16

**Authors:** Mónika Gabriella Kovács, Ana Isabel Antunes Goncalves, Zoltán Varga, Réka Losonczi, Klaudia Kupecz, Dávid Volford, Szonja Polett Pósa, Renáta Minorics, Zsolt Galla, Karin Zins, Anita Sejben, András Kriston, Ferenc Kovács, Péter Horváth, Péter Monostori, Gábor Cserni, István Zupkó, Bruno K. Podesser, Eytan R. Barnea, Zsuzsanna Kahán, Attila Kiss, Peter Pokreisz, Márta Sárközy

**Affiliations:** 1 Department of Biochemistry and Interdisciplinary Center of Excellence, Albert Szent-Györgyi Medical School, University of Szeged, Szeged, Hungary; 2 Center for Biomedical Research and Translational Surgery, Medical University of Vienna, Vienna, Austria; 3 Department of Oncotherapy, Albert Szent-Györgyi Medical School, University of Szeged, Szeged, Hungary; 4 Department of Pathophysiology, Albert Szent-Györgyi Medical School, University of Szeged, Szeged, Hungary; 5 Institute of Pharmacodynamics and Biopharmacy, University of Szeged, Szeged, Hungary; 6 Metabolic and Newborn Screening Laboratory, Department of Pediatrics, Albert Szent-Györgyi Medical School, University of Szeged, Szeged, Hungary; 7 Center for Anatomy and Cell Biology, Medical University of Vienna, Vienna, Austria; 8 Department of Pathology, Albert Szent-Györgyi Medical School, University of Szeged, Szeged, Hungary; 9 Synthetic and Systems Biology Unit, HUN-REN Biological Research Centre, Szeged, Hungary; 10 Single-Cell Technologies Ltd, Szeged, Hungary; 11 Institute for Molecular Medicine Finland (FIMM), University of Helsinki, Helsinki, Finland; 12 Institute of AI for Health, Helmholtz Zentrum München, Neuherberg, Germany; 13 S.I.E.P. The Society for the Investigation of Early Pregnancy, New York, NY, United States; 14 Obstetrics, Gynecology & Reproductive Sciences, Miller School of Medicine, University of Miami, Miami, FL, United States; 15 BioIncept, LLC (*sPIF Proprietary), New York, NY, United States; 16 Faculty of Health and Social Sciences, Gál Ferenc University, Gyula, Hungary

**Keywords:** fibrosis, heart failure, inflammation, nitro-oxidative stress, radiation protection, radiation-induced heart damage, synthetic preimplantation factor (sPIF)

## Abstract

**Background:**

Radiation-induced heart disease (RIHD) is a potential side effect of radiotherapy (RT) for thoracic tumors and breast cancers, often causing diastolic dysfunction, left ventricular hypertrophy (LVH), and fibrosis. Preimplantation factor (PIF), an embryonic immunomodulatory peptide, has shown protective effects against acute radiation syndrome. We investigated whether synthetic PIF (sPIF) could mitigate RIHD development in rats and tumor cell proliferation in human breast cancer cell lines.

**Methods:**

Adult male Sprague-Dawley rats were allocated into three experimental groups: vehicle-treated control, vehicle-treated RT (50 Gy, focused on the heart), and RT treated with sPIF, monitored at 1, 3, and 15 weeks. Endpoint assessments included transthoracic echocardiography, histopathology, qPCR, Western blotting, and UHPLC–MS/MS in the left ventricular (LV) samples. The effects of sPIF on hypertrophy, fibrosis, and proliferation were investigated in H9c2 cardiomyoblasts, human ventricular cardiac fibroblasts (HVCFs), and human breast carcinoma cells, respectively.

**Results:**

sPIF decreased LV expression of inflammatory markers at week 3 and attenuated RT-induced LVH and fibrosis, with reduced nitrotyrosine accumulation at week 15. sPIF suppressed angiotensin II–induced hypertrophy in H9c2 cells, inhibited transforming growth factor-*β*1 (TGF*β*1)–induced collagen-1 expression in HVCFs, and enhanced the anti-proliferative response to RT in human breast carcinoma cells.

**Conclusions:**

These findings suggest the potential of sPIF as a cardioprotective agent for the management of RIHD and warrant further mechanistic investigations.

## 1 Introduction

The significant improvement in oncologic treatment modalities has led to increased long-term survival among cancer patients (1). High-energy ionizing radiation in the form of radiotherapy (RT) is an important treatment approach for breast cancer and thoracic malignancies, including lung and esophageal cancers, Hodgkin's lymphoma, and thymoma (2, 3). Cardiovascular complications are the most common non-malignant causes of death among cancer survivors who received RT (4, 5). This risk is further exacerbated by chemotherapy in multimodal oncologic regimens and by preexisting comorbidities, including diabetes mellitus, hypertension, dyslipidemia, and obesity (6).

While RT is still an effective treatment for breast cancer, it potentially damages the surrounding healthy tissues and organs, including the heart, and results in radiogenic sequelae depending on the applied technique and dose. Hence, RT contributes to the development of radiation-induced heart disease (RIHD) (7, 8). It is a progressive and maladaptive broad spectrum of cardiac pathologies, including pericarditis, LVH, conduction system abnormalities, coronary artery disease, valvular heart diseases, and cardiomyopathy, which may ultimately lead to heart failure (HF) with either preserved or reduced ejection fraction (HFpEF and HFrEF, respectively) (5, 9–11). Notably, RT-related cardiac fibrosis is a late complication, typically appearing 10 or more years after irradiation, particularly in cases with a cumulative dose exceeding 30 Gy (6). Moreover, LV diastolic dysfunction has been more widely reported and linked to RT than systolic dysfunction (6).

RT causes simultaneous injury to the myocardium (i.e., diffuse injury) and to the cardiac macro- and microvasculature (7, 8, 12). RT-induced nitro-oxidative stress directly initiates RIHD development by damaging proteins, lipids, and DNA. Injury to cardiomyocytes (CMs), endothelial cells, and other cells ultimately leads to cell death and inflammation (7, 13, 14). As a compensatory mechanism for cell loss, surviving CMs may undergo hypertrophy during the early chronic phase of RIHD (7, 8). If compensatory mechanisms fail to mitigate the damage, chronic inflammation initiates fibrosis progression and endothelial dysfunction, which become the predominant pathological features driving RIHD (13, 14). The precise molecular mechanisms involved in the development of RIHD remain largely elusive. Additionally, no clinical evidence supports the effectiveness of standard cardioprotective medications, comprising statins or angiotensin-converting enzyme inhibitors, in managing RIHD (6). Therefore, preventive and therapeutic strategies are urgently needed, along with novel compounds that preserve the anti-cancer efficacy of RT.

The native preimplantation factor (PIF) is an evolutionarily conserved 15-amino-acid peptide secreted by viable mammalian embryos and is a critical factor in maternal tolerance of the fetal allograft (15). PIF provides integrated local and systemic immune protection, transplant acceptance, and cell- and organ-controlled growth (16, 17). It is involved in promoting maternal/host and embryo/graft acceptance (16, 18). PIF supports embryo implantation by modulating cell proliferation and apoptosis, and it exhibits immunomodulatory, anti-inflammatory, and regenerative properties (19, 20). In human trophoblast cells, PIF decreased the phosphorylation of p53 at Ser-15, enhanced the B-cell lymphoma-2 (BCL2) expression, and reduced the BCL2-associated X protein (BAX) and BCL2 homologous antagonist killer (BAK) transcript levels (21). The lessons learned from pregnancy were translated to non-pregnancy applications, including neurologic diseases, atherosclerosis, and radiation protection (22–24).

Indeed, a synthetic PIF (sPIF) analog containing the same amino acid sequence as native PIF showed protective effects against neuroinflammation in murine models of experimental autoimmune encephalomyelitis and hypoxic-ischemic brain injury (25). In a type 1 diabetes mellitus animal model, sPIF reduced oxidative stress, prevented protein misfolding and aggregation, pancreatic islet apoptosis, and inhibited neutrophil invasion and inflammation (26). sPIF mitigated cardiac and other muscle fibrosis in a Duchenne muscular dystrophy model (27). It protected against acute radiation syndrome (ARS) by preserving the global immune system after lethal radiation (100% survival vs. 0% in controls) (24). Also, after sublethal radiation, sPIF completely restored colonic architecture, protected the jejunum, and reduced systemic inflammation in ARS (24). Following lethal radiation and semi-allogeneic bone marrow transplantation, low-dose, short-term sPIF reduced mortality 6-fold at 4 months, protecting the skin, liver, colon, and systemic immunity in ARS (24). Mechanistically, sPIF primarily targets protein disulfide isomerase/thioredoxin (PDI/T) and heat shock proteins (HSPs), thereby reducing oxidative stress and protein misfolding and preventing cellular damage (28). These targeted protective mechanisms are key in addressing RT-induced organ damage. Thus, sPIF is being pursued as a potential therapeutic agent for RT-induced cardiac dysfunction.

Therefore, the present study aimed to *i)* investigate the potential radioprotective properties of sPIF in a rat model of RIHD (Figure 1); *ii*) elucidate several underlying cardioprotective mechanisms using H9c2 cardiomyoblasts and HVCFs; and *iii*) test the effects of sPIF on the proliferation of human breast cancer cell lines exposed to ionizing radiation.

**Figure 1:**
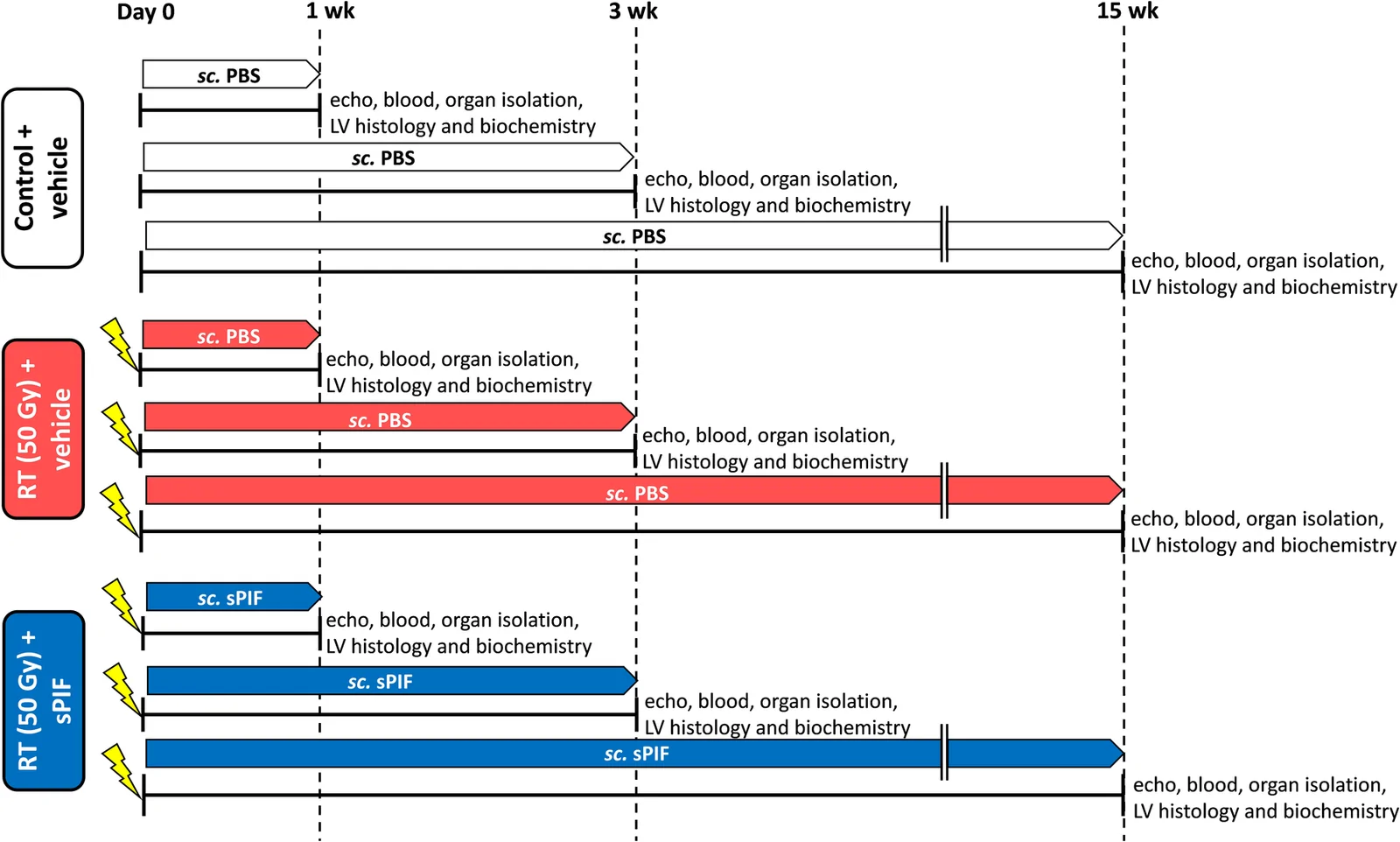
Experimental design. A total of 69 rats were randomized based on body weight (*n* = 7-8) into three separate experimental groups and monitored for 1, 3, or 15 weeks. i) Vehicle-treated control groups received phosphate-buffered saline (PBS, sc. 2 mL/kg/day in the 1-week group and daily for 2 weeks and then twice weekly in the 3- and 15-week groups). ii) RT (50 Gy focused on the heart, PBS under the same dosing regimen as the control groups). iii) sPIF-treated RT groups received synthetic preimplantation factor (sc. 1 mg/kg/day for 1 week and daily for 2 weeks and then twice weekly in the 3- and 15-week groups). The vehicle-treated control groups contained 7, 8, and 7 animals at weeks 1, 3, and 15, respectively. The vehicle-treated RT groups had 8 rats at each time point. The sPIF-treated groups had 7 animals at each time point. At the end of the different follow-up times, echocardiography (echo) was performed, and then the hearts, lungs, and tibias were excised under anesthesia. Left ventricular (LV) samples were prepared for histological and biochemical analyses, including quantitative polymerase chain reaction (qPCR), Western blot, and ultra-high-performance liquid chromatography-tandem mass spectrometry (UHPLC-MS/MS). In all figures, white bars indicate the vehicle-treated control (control + vehicle) groups, red bars the vehicle-treated radiotherapy (RT + vehicle) groups, and blue bars the sPIF-treated radiotherapy (RT + sPIF) groups.

## 2 Methods

### 2.1 Ethics approval

This investigation conformed to the EU Directive 2010/63/EU and was approved by the regional Animal Research Ethics Committee of Csongrád County (project license: XV./800/2019, date of approval: 30 May 2019) and the University of Szeged, Hungary.

### 2.2 Animals and experimental groups

A total of 69 male Sprague-Dawley rats (220–300 g, 6–7 weeks old, Charles-Rivers Laboratories, Germany) were allocated to separate experimental cohorts monitored for 1, 3, or 15 weeks (*n* = 7–8 per group, Figure 1). At each time point, animals were randomized based on body weight and allocated to three groups. Treatments were administered as follows: *i*) vehicle-treated control groups received phosphate-buffered saline (PBS). Administration was subcutaneous (*sc.*), 2 mL/kg/day for 1 week. In the 3- and 15-week experiments, PBS was given daily for 2 weeks, and then twice weekly thereafter. *ii*) vehicle-treated RT groups received PBS under the same dosing regimen as the vehicle-treated control groups. *iii*) sPIF-treated RT groups received sPIF (*sc.* 1 mg/kg/day for 1 week). In the 3- and 15-week experiments, sPIF was administered daily for 2 weeks and then twice weekly thereafter. The sPIF (Piramal Pharma Substances, Lexington, KY, USA) was prepared and purified to greater than 95% purity, documented by mass spectrometry as described previously (29). To eliminate oxidation, sPIF was dissolved in deoxygenated PBS to a final volume of 2 mL/kg (Figure 1). The vehicle-treated control groups contained 7, 8, and 7 animals at weeks 1, 3, and 15, respectively. The vehicle-treated RT groups had 8 rats at each time point. The sPIF-treated groups had 7 animals at each time point. No animals died during the experiment. The animals were housed in individually ventilated cages (Tecniplast Sealsafe IVC system, Buguggiate, Italy) under a 12/12-hours light/dark cycle in a temperature-controlled room (22 ± 2 °C; relative humidity 55 ± 10%) with access to standard rat chow and tap water *ad libitum*.

### 2.3 Experimental setup

Cardiac morphology and function were assessed by transthoracic echocardiography under isoflurane anesthesia at the endpoint of each experiment (Figure 1). At the end of the different follow-up times, the rats were anesthetized with sodium pentobarbital (Euthasol; 40 mg/kg, *ip.*; Produlab Pharma b.v., Raamsdonksveer, The Netherlands), and the abdominal cavity was opened to collect 1- 1.5 mL of blood from the aorta. Rats were euthanized by overdosing with sodium pentobarbital (100 mg/kg, *ip.*). The heart, lungs, and tibias were excised, and the organs were perfused with Krebs-Henseleit solution to remove residual blood. The total heart, LV, and wet lung weights, as well as the tibia length, were measured, and the LVs were transversely sectioned from the mid-papillary to the basal level. The basal parts were fixed in 4% buffered formalin for histological analysis. The apical parts of the LVs were freshly frozen in liquid nitrogen and stored at −80 °C until further biochemical measurements, including qPCR (see the list of primers in Supplementary Table 1), Western blot, and ultra-high-performance liquid chromatography-tandem mass spectrometry (UHPLC-MS/MS, Figure 1). The detailed methods are described in the Supplemental Material.

### 2.4 Heart irradiation

Heart irradiation with a single dose of 50 Gy was carried out in both the vehicle-treated and sPIF-treated RT groups, as described previously in our dose-finding and treatment experiments (30–32). Before irradiation, rats were anesthetized with sodium pentobarbital (Euthasol, i.p., 40 mg/kg, Produlab Pharma b.v., Raamsdonksveer, The Netherlands) and then fixed in a supine position on a flat surface couch. The irradiation plan was based on a 3D model (Eclipse v.13.6 RT, Varian Oncology Systems, Palo Alto, CA, USA) generated in the planning system. The dose was prescribed to the geometric center of the heart. To improve heart coverage and lung protection, a 6 MeV electron beam was delivered through a circular aperture with a diameter of 2 cm. According to quality control dosimetry measurements, dose homogeneity within the volume of the heart remained within ±5%. After verifying the animal's position with a built-in electronic portal imaging device, the radiation dose was delivered with a Varian TrueBeam STx linear accelerator (Varian Oncology Systems, Palo Alto, CA, USA).

### 2.5 sPIF administration

In independent toxicology studies, the “no observed adverse effect level” (NOAEL) of sPIF was found to be 400 mg/kg in mice. (NCATS/NIH) In preclinical models of autoimmune diseases and transplantation, doses of 0.1-1 mg/kg of sPIF had the maximum efficacy (17, 24, 26, 33). Moreover, sPIF safety at 0.1–1.0 mg/kg given *sc.* was confirmed in an FDA clinical trial for autoimmune liver disease (34). In our previous dose-finding experiments, there were no significant differences in the fibrosis- and hypertrophy-limiting effects of sPIF assessed by histology and echocardiography between 1 and 1.5 mg/kg sPIF doses in a similar rat model of RIHD irradiated with 50 Gy and followed up for 19 weeks (35). Therefore, we used the maximum sPIF dose (1 mg/kg) in our present study. The initial daily *sc.* treatment period was based in part on prior work in an experimental autoimmune encephalomyelitis model, in which sPIF was initially administered during active neurological disease followed by episodic administration to prevent paralysis recurrence (25, 36). This approach was adapted to the longer course of RIHD by administering sPIF daily for the first 2 weeks and twice weekly thereafter in our present 3- and 15-week experiments. The effects of sPIF on RIHD were analyzed at 1, 3, and 15 weeks, reflecting the development of RT-induced cardiac pathology, similar to our previous study (32).

### 2.6 Cell culture experiments

*In vitro* cellular experiments were conducted to investigate the inhibitory effects of sPIF on hypertrophy in H9c2 cardiomyoblasts (American Type Culture Collection, ATCC, USA), the anti-fibrotic phenotype in HVCFs (Lonza, Switzerland), and tumor cell proliferation in three different human breast carcinoma cell lines (MCF-7, T47D, and MDA-MB-231; ECCAC, Salisbury, UK) exposed to radiation (4 Gy). The detailed methods are described in the Supplemental Material.

### 2.7 Statistical analyses

All analyses were performed with GraphPad Prism Software (version 8.01, GraphPad Software Inc., Boston, MA, USA). The normality of data distribution was tested using the Shapiro–Wilk test. Two-way analysis of variance (ANOVA) was used for comparisons among groups within each time point in the animal experiments, and one-way ANOVA was used in the cell culture experiments. In cases of significant differences between the groups, multiple pairwise comparisons were adjusted using the Holm-Sidak correction in the *post hoc* test. In cell culture experiments in breast cancer cell lines, Tukey's *post hoc* test was used. All values are presented as mean ± SEM, and a *p*-value <0.05 was considered statistically significant. The descriptive statistics for the animal experiments are presented in Supplementary Tables 2 and 3, with significant differences (*p* < 0.05) highlighted in red.

## 3 Results

### 3.1 Systemic effects of RT in the rat model of RIHD

At week 1, there were no significant differences in body weight, tibia length, or the ratios of body, total heart, LV, and lung weights to tibia length among the groups (Figures 2A–F). At week 3, the body weights were significantly lower in both RT groups compared to the vehicle-treated control group (Figure 2A). Interestingly, at week 3, the wet lung weight-to-tibia length ratios were markedly higher in both RT groups compared to the vehicle-treated control group, suggesting the presence of pulmonary edema at week 3 (Figure 2F). At week 15, body weight and tibia length were significantly lower in the RT group compared to the vehicle-treated control group (Figures 2A,B). The sPIF treatment prevented RT-induced growth and developmental retardation, as both body weight and tibia length were markedly higher in the RT + sPIF group than in the vehicle-treated RT group (Figures 2A,B). However, there were no significant differences in the body weight-to-tibia length ratios among the groups at week 15 (Figure 2C). Moreover, at week 15, the total heart and LV weights to tibia length ratios were markedly reduced in both RT groups compared to the vehicle-treated control group, suggesting myocardial cell loss as a potential late consequence of RT in the heart tissue (Figures 2D,E).

**Figure 2:**
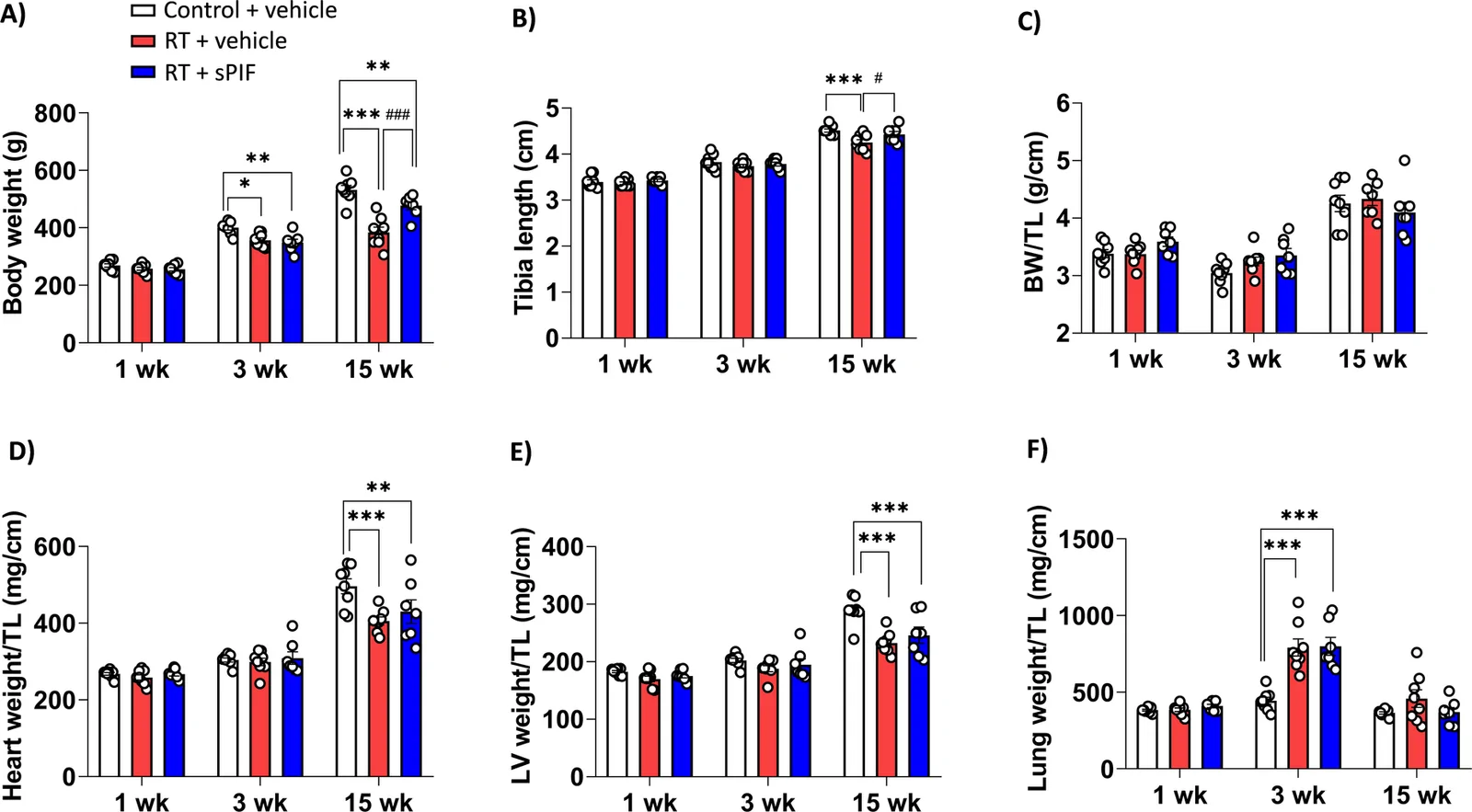
The effects of synthetic preimplantation factor (sPIF) on the morphometric parameters during the development of radiation-induced heart disease. **(A)** Body weight, **(B)** tibia length, **(C)** body weight to tibia length, **(D)** heart weight to tibia length, **(E)** left ventricular (LV) weight to tibia length, and **(F)** lung weight to tibia length ratios at weeks 1, 3, and 15. Values are presented as mean ± SEM, *p < 0.05, **p < 0.01, and ***p < 0.001 *vs*. vehicle-treated control, ^#^*p* < 0.05, and ^####^*p* < 0.001 *vs*. vehicle-treated RT, *n* = 7-8, two-way ANOVA, Holm-Sidak *post hoc* test.

### 3.2 sPIF treatment limited LVH 15 weeks post-irradiation

No significant differences were observed in cardiac morphology or functional parameters across the groups 1 week after irradiation (Figures 3A–I, 4A–F, Supplementary Figures 1A–E, 2A–D, 3A–E, and 4A–C). At week 3, the sPIF-treated RT group exhibited marked reductions in the left ventricular end-diastolic diameter (LVEDD) and left ventricular end-systolic diameter (LVESD) compared to the vehicle-treated control or RT groups (Figures 3A,E). These changes were accompanied by increased long-axis fractional shortening (FS) in the RT + sPIF group (Supplementary Figure 2B). At week 15, the RT-induced initial cell loss was likely compensated by the hypertrophy of the surviving cardiomyocytes (Figures 3A,D and Supplementary Figures 1A–E). Indeed, the significantly increased septal, posterior, and anterior wall thicknesses, as well as relative wall thickness (RWT), suggest the development of concentric LVH in the vehicle-treated RT group compared with the vehicle-treated control group (Figures 3A,F; Supplementary Figures 1A–E). Moreover, the LVEDD and LVESD were significantly decreased due to the compensatory LVH in both irradiated groups (Figure 3E and Supplementary Figure 2A). The long axis FS were markedly higher in both irradiated groups compared to the vehicle-treated control group, probably due to the more pronounced changes of the LVESD compared to LVEDD (Supplementary Figures 2A,B, Figure 3E). At week 15, sPIF treatment partially reversed irradiation-induced concomitant LVH, reflected by significant reductions in wall thicknesses and RWT as well as improvement in the LVEDD, LVESD, and FS (Figures 3A,D–F, Supplementary Figure 1A–E, and 2A,B).

**Figure 3:**
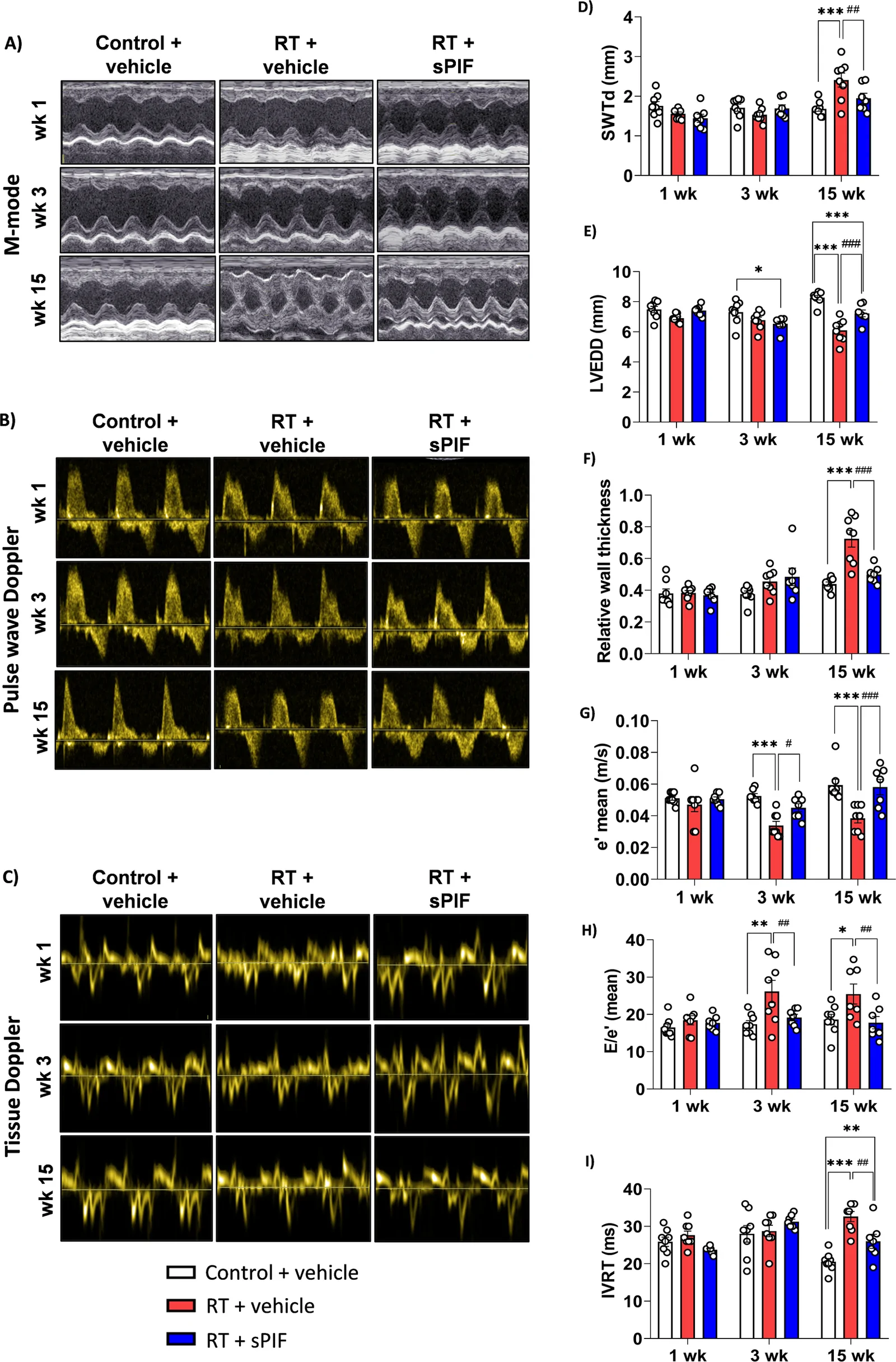
The effects of synthetic preimplantation factor (sPIF) on echocardiographic parameters during the development of radiation-induced heart disease. Representative **(A)** M-mode, **(B)** pulsed wave Doppler, and **(C)** tissue Doppler images, **(D)** septal wall thickness in diastole (SWTd), **(E)** left ventricular end-diastolic diameter (LVEDD), **(F)** relative wall thickness [(2xPWTd)/LVEDD], **(G)** diastolic mitral annulus velocity (e’) calculated as the mean value of septal and lateral e’ (mean e’ = ([septal e’+lateral e’]/2), **(H)** ratio of early mitral flow velocity **(E)** and e’, **(I)** isovolumic relaxation time (IVRT) at weeks 1, 3, and 15. Values are presented as mean ± SEM, *p < 0.05, **p < 0.01, and ***p < 0.001 *vs*. vehicle-treated control, ^#^*p* < 0.05, ^##^*p* < 0.01, and ^####^*p* < 0.001 *vs*. vehicle-treated RT, *n* = 7-8, two-way ANOVA, Holm-Sidak *post hoc* test. Maximal scale depth in M-mode images: 3-4 cm, maximal scale velocity in Doppler images: 1 m/s, and maximal scale velocity in tissue Doppler images: 10 cm/s.

**Figure 4:**
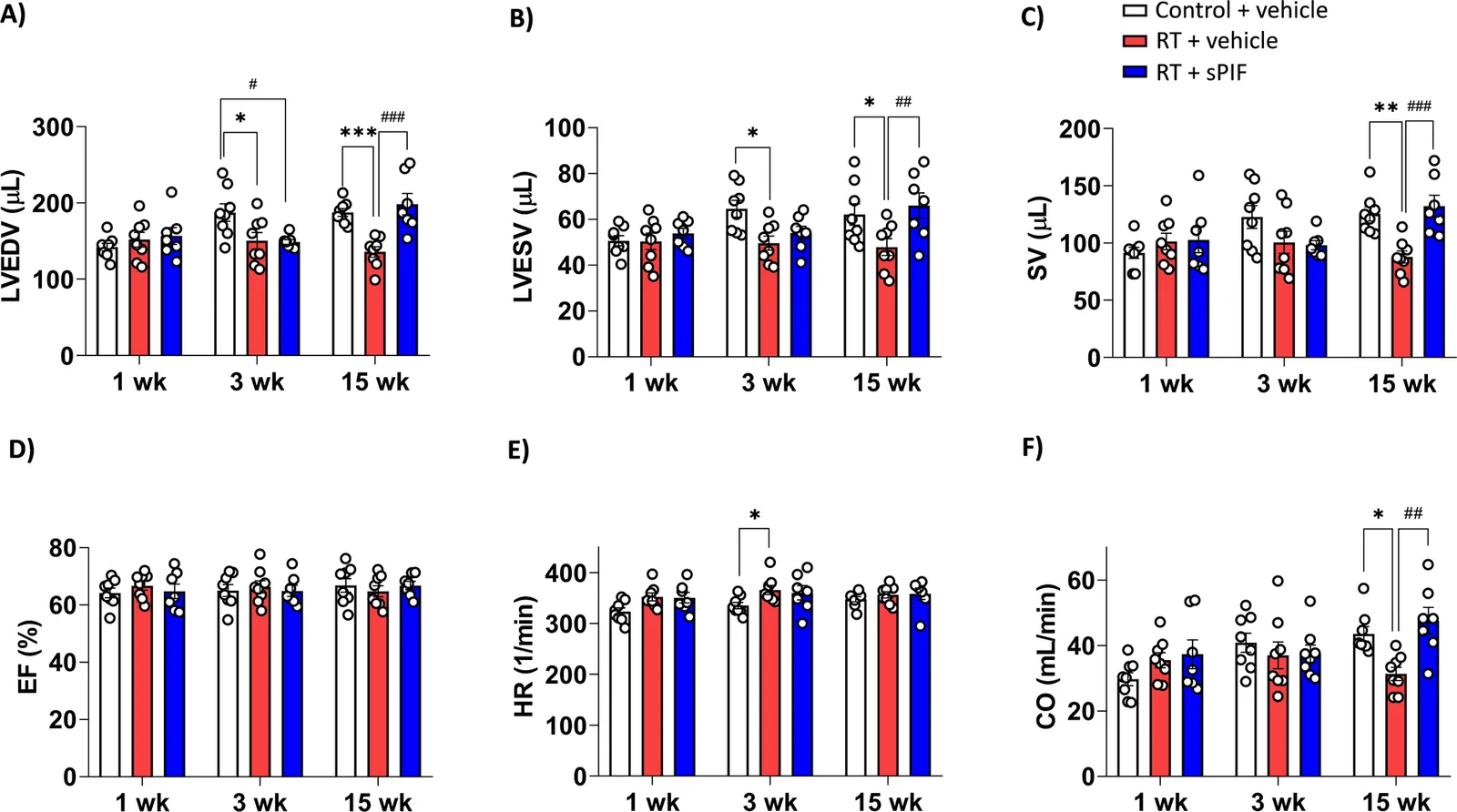
The effects of synthetic preimplantation factor (sPIF) on LV volumes and hemodynamic parameters measured by echocardiography during the development of radiation-induced heart disease. **(A)** Left ventricular end-diastolic volume (LVEDV, **(B)** left ventricular end-systolic volume (LVESV), **(C)** stroke volume (SV), **(D)** ejection fraction (EF), **(E)** heart rate (HR), and **(F)** cardiac output (CO). Values are presented as mean ± SEM, *p < 0.05, **p < 0.01, and ***p < 0.001 *vs*. vehicle-treated control, ^#^*p* < 0.05, ^##^*p* < 0.01, and ^####^*p* < 0.001 *vs*. vehicle-treated RT, *n* = 7-8, two-way ANOVA, Holm-Sidak *post hoc* test.

### 3.3 sPIF treatment ameliorated the diastolic dysfunction at 3- and 15-week post-irradiation

At week 1, there was no significant difference in the mitral annulus velocity (e’) or the mitral valve early flow velocity (E) to e’ ratio (E/e’), and isovolumic relaxation time (IVRT) between the groups (Figures 3B–G–I, Supplementary Figures 3A–E), probably owing to the lack of severe morphologic changes at this early time point of RIHD. In contrast, at week 3, the septal, lateral, and mean e’ were markedly reduced, and the E/e’ (calculated with septal, lateral, or mean e’) was significantly higher in the vehicle-treated RT group compared to the vehicle-treated control group (Figures 3G,H, Supplementary Figures 3A–E). This RT-induced diastolic dysfunction was markedly reduced by the sPIF treatment compared to the vehicle-treated RT group at week 3, as indicated by the markedly increased e’ (mean) and decreased E/e’ (calculated with lateral or mean e’) compared to the vehicle-treated RT group (Figures 3G,H, Supplementary Figures 3D,E). At week 15, the E was significantly decreased, the septal, lateral, and mean e’ were markedly reduced, and the E/e’ ratio calculated with septal or mean e’ was significantly elevated in the vehicle-treated RT group compared to the vehicle-treated control group, indicating diastolic dysfunction (Figures 3G,H, Supplementary Figures 3A–E). Moreover, the IVRT was significantly prolonged in the vehicle-treated RT group compared to the vehicle-treated control group, further supporting the development of diastolic dysfunction at week 15 (Figure 3I). There were no marked differences in the isovolumic contraction time (IVCT) and ejection time (ET) among the groups at any follow-up time point (Supplementary Figures 4A,B). Consequently, the prolonged IVRT resulted in a significant increase in the Tei index in the vehicle-treated RT group compared to the vehicle-treated control group at week 15 (Supplementary Figure 4C). Interestingly, there was no significant difference in the E between the RT + sPIF and vehicle-treated control groups at week 15 (Figure 3B and Supplementary Figure 3A). Notably, the septal, lateral, and mean e’, the E/e’ ratio (calculated with septal or mean e’), IVRT, and Tei index were significantly improved by sPIF treatment compared to the vehicle-treated RT group (Figures 3G–I, Supplementary Figures 3B–E, 4A–C).

### 3.4 sPIF treatment ameliorated the reduction of cardiac output at 15 weeks post-irradiation

At week 1, there were no significant differences in the left ventricular end-diastolic and end-systolic volumes (LVEDV and LVESV, respectively), stroke volume (SV), ejection fraction (EF), heart rate (HR), and cardiac output (CO) (Figures 4A–F). At week 3, LVEDV was markedly reduced in both irradiated groups, and LVESV was decreased in the vehicle-treated RT group compared with the vehicle-treated control group (Figures 4A,B). As compensation for decreased LV volumes, the HR was markedly increased in the vehicle-treated RT group compared to the vehicle-treated control group at week 3 (Figure 4E). In response to sPIF treatment, there were no significant differences in the LVEDV, LVESV, and HR compared to the vehicle-treated control group at week 3 (Figures 4A–E). At week 15, concentric LVH resulted in markedly reduced LVEDV and LVESV, leading to a significantly lower SV in the vehicle-treated RT group compared to the vehicle-treated control group (Figures 4A–C). The proportionally decreased LVEDV and LVESV resulted in preserved EF in the vehicle-treated RT group compared with the vehicle-treated control group at week 15 (Figure 4D). The HR remained unchanged in the vehicle-treated RT group, leading to a markedly reduced CO compared with the vehicle-treated control group at week 15 (Figures 4E,F). Importantly, at 15 weeks post-irradiation, the sPIF treatment significantly normalized LVEDV, LVESV, SV, and CO compared with the vehicle-treated RT group (Figures 4A–C,F).

### 3.5 sPIF treatment alleviated CM hypertrophy at the cellular level and prevented the adult myosin heavy chain replacement by fetal isoform 15 weeks after RT

At weeks 1 and 3 post-irradiation, there were no significant differences in the CM perimeters and cross-sectional areas between the groups (Figures 5A–C). At week 1, the LV expressions of adult myosin heavy chain *α*-isoform (*Myh6*) and the fetal myosin heavy chain *β*-isoform (*Myh7*) were markedly elevated in the sPIF-treated RT group compared to the vehicle-treated RT group (Figures 5D,E). At week 1, *Myh7* and the *Myh7/Myh6* ratio tended to increase in the vehicle-treated RT group compared to the vehicle-treated control group. The sPIF treatment significantly increased both the *Myh7* expression and the *Myh7/Myh6* ratio compared to the vehicle-treated control group (Figures 5E,F). At week 3, LV *Myh6* expression was markedly lower in the sPIF-treated RT group than in the vehicle-treated control group (Figure 5D). At week 3, sPIF treatment significantly reduced *Myh7* overexpression and the *Myh7/Myh6* ratio (Figures 5E,F). At week 15, CMs showed significantly increased perimeter and cross-sectional area in the vehicle-treated RT group compared with the vehicle-treated control group, confirming the development of compensatory hypertrophy at the cellular level (Figures 5A–C). Notably, there were no marked differences in CM perimeters and cross-sectional areas between the sPIF-treated RT and vehicle-treated control groups at week 15 (Figures 5A–C). At week 15, the *Myh6* expression was significantly reduced, the *Myh7* expression tended to increase, and the *Myh7/Myh6* ratio was markedly higher in the vehicle-treated RT group than in the vehicle-treated control group, indicating molecular signs of LVH development (Figures 5D–F). At week 15, sPIF preserved healthy adult myosin heavy chain isoform content compared with the vehicle-treated RT group (Figures 5D–F).

**Figure 5:**
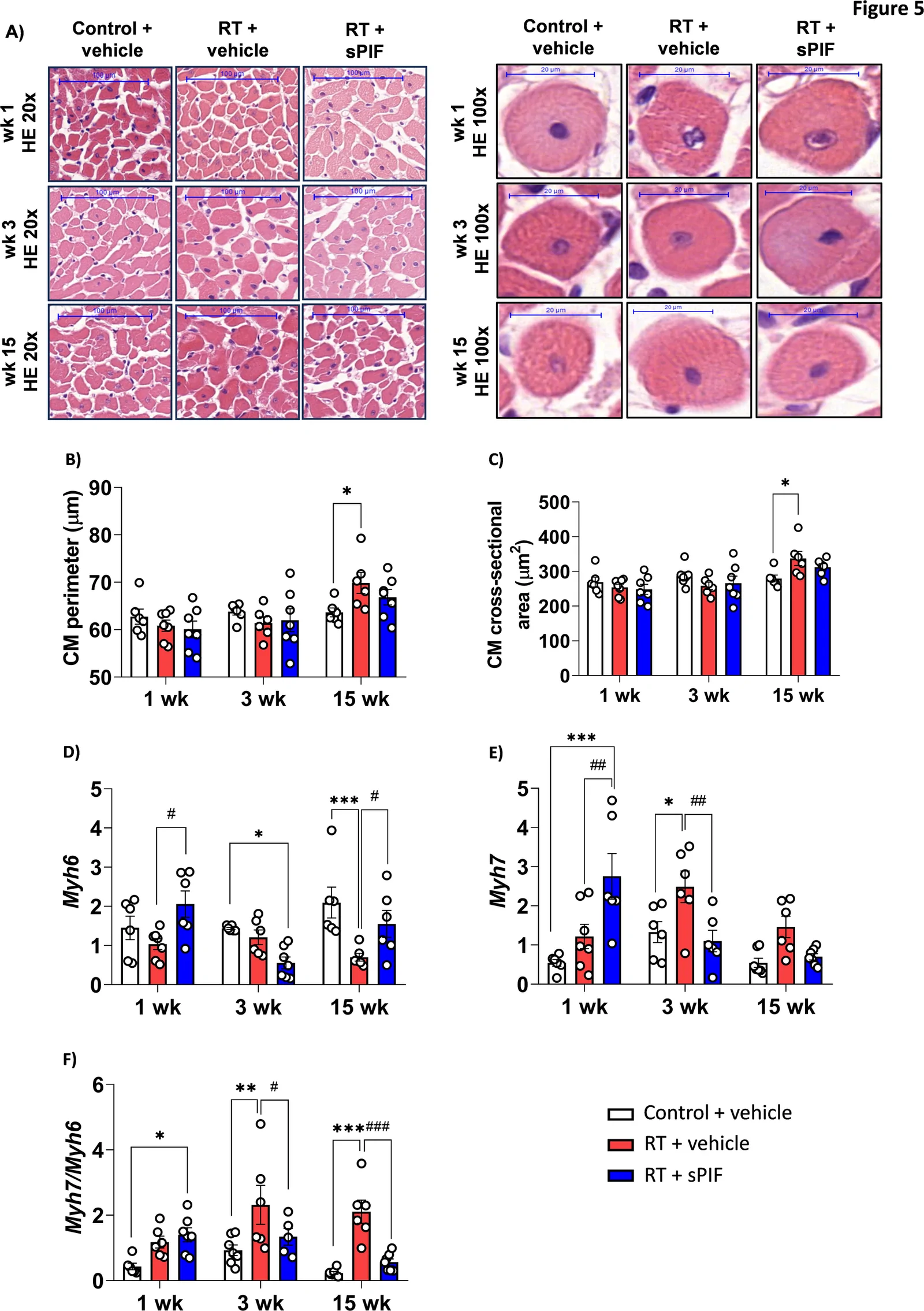
The effects of synthetic preimplantation factor (sPIF) on the histological signs and molecular markers of cardiac hypertrophy in radiation-induced heart disease. **(A)** Representative hematoxylin-eosin (HE)-stained slides of left ventricles (20x and 100x magnifications), **(B)** cardiomyocyte (CM) perimeters and **(C)** cross-sectional areas, **(D)** left ventricular expression of the *α*-myosin heavy chain (*Myh6*) and **(E)** the *β*-myosin heavy chain (*Myh7*), **(F)** *Myh7/Myh6* ratio at weeks 1, 3, and 15. Values are presented as mean ± SEM, *p < 0.05, **p < 0.01, ***p < 0.001 *vs*. vehicle-treated control, ^#^*p* < 0.05, ^##^*p* < 0.01, and ^####^*p* < 0.001 *vs*. vehicle-treated RT, *n* = 6-7, two-way ANOVA, Holm-Sidak *post hoc* test.

### 3.6 sPIF treatment reduced the RIHD-associated cardiac fibrosis

At week 1, LV connective tissue growth factor (*Ctgf*) expression did not differ significantly among groups; however, transforming growth factor-*β*1 (*Tgfb*) was markedly overexpressed after sPIF treatment compared with the vehicle-treated control or vehicle-treated RT groups (Figures 6A,B). In contrast, there were no marked differences in the LV SMAD2/3 protein levels and the LV fibrosis among the groups at week 1 (Figures 6C–E). The LV expression of the collagenase matrix metalloprotease 2 *(Mmp2)* tended to increase in the vehicle-treated RT group and elevated significantly in the sPIF-treated RT group compared to the vehicle-treated control or the vehicle-treated RT groups at week 1 (Figure 6F). The LV expression of A-type natriuretic peptide (*Nppa*), a myocardial stretch marker, was not different among the groups at week 1 (Figure 6F). At week 3, the sPIF treatment lowered the LV *Tgfb* expression compared to the vehicle-treated control group (Figure 6B). It markedly reduced the LV overexpression of *Mmp2* compared to the vehicle-treated RT group (Figure 6F). At week 15, the sPIF treatment significantly reduced the LV expression of *Ctgf* compared to the vehicle-treated RT group (Figure 6A). There were no marked differences in the LV *Tgfb* expressions among the groups (Figure 6B). In contrast, the sPIF treatment significantly reduced LV protein levels of SMAD2/3 compared to the vehicle-treated RT group (Figure 6C). Notably, sPIF significantly decreased LV fibrosis at week 15 (Figures 6D,E). LV *Mmp2* expression did not differ significantly between groups at week 15 (Figure 6F). Moreover, sPIF treatment significantly mitigated *Nppa* overexpression compared with the vehicle-treated RT group at week 15 (Figure 6G).

**Figure 6:**
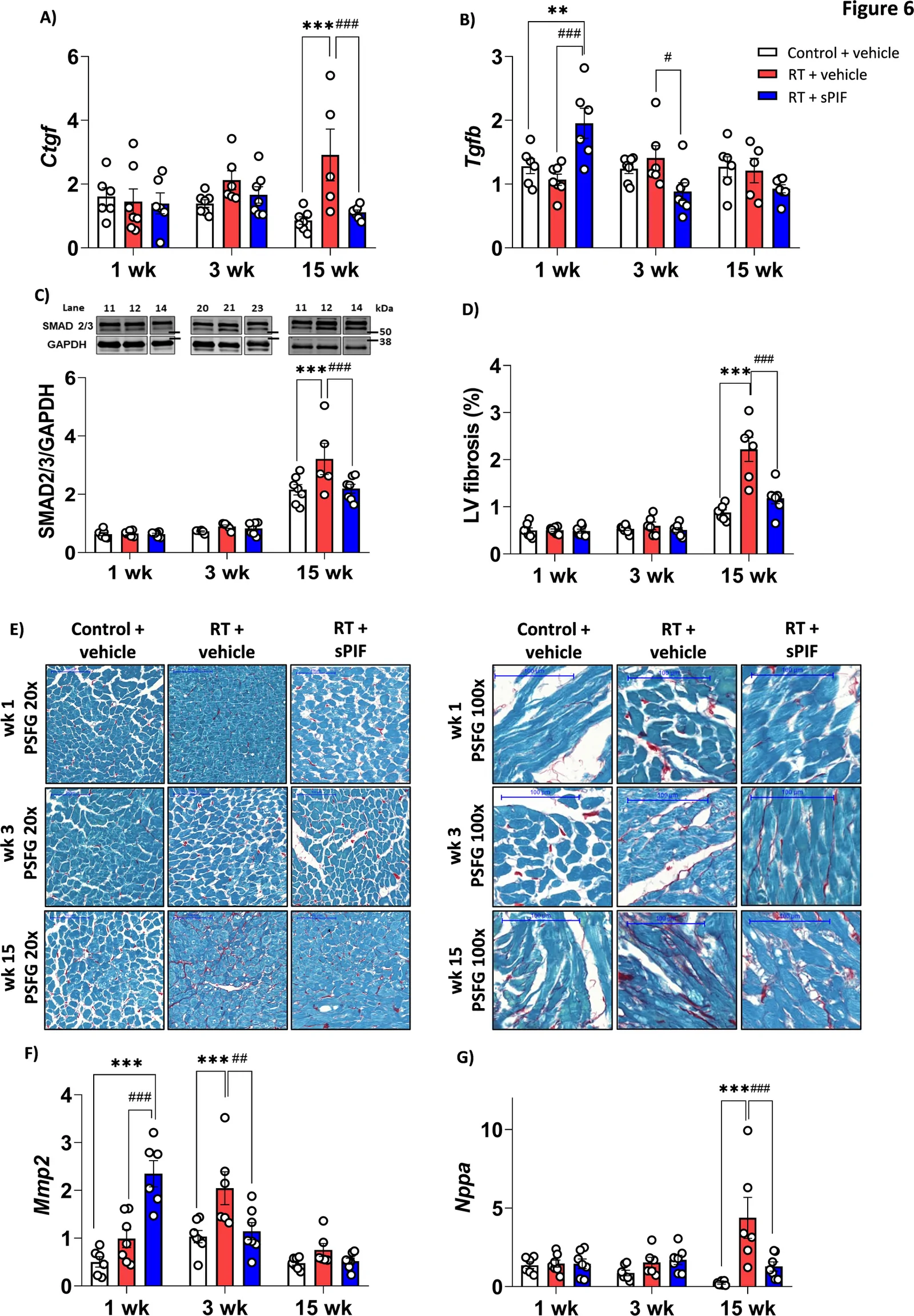
The effects of synthetic preimplantation factor (sPIF) on the molecular and histologic signs of fibrosis development in radiation-induced heart disease. **(A)** Left ventricular (LV) transcript levels of connective tissue growth factor (*Ctgf*), **(B)** transforming growth factor-*β*1 (*Tgfb*), **(C)** LV protein levels of mothers against decapentaplegic homolog 2 (SMAD2/3) normalized to glycerin aldehyde-3 phosphate dehydrogenase (GAPDH), **(D)** LV fibrosis, **(E)** representative picrosirius red and fast green (PSFG) stained slides (20x and 100x magnifications), **(F)** transcript levels of matrix metalloprotease-2 (*Mmp2*) and **(G)** A-type natriuretic peptide (*Nppa*) at weeks 1, 3, and 15. Values are presented as mean ± SEM, **p < 0.01 and, ***p < 0.001 *vs*. vehicle-treated control, ^#^*p* < 0.05, ^##^*p* < 0.01, and ^####^*p* < 0.001 *vs*. vehicle-treated RT, *n* = 6-7, two-way ANOVA, Holm-Sidak *post hoc* test. Western blot images were captured using the Odyssey CLx and exported with Image Studio 5.2.5. Black lines on the Western blot images show the grouping of the cropped blots. The full-length Western blots are presented in Supplementary Figures S12-16.

### 3.7 sPIF treatment decreased the overexpression of cardiac inflammation markers in RIHD after three weeks

At week 1, the LV transcript levels of interleukin-1 (*Il1*) tended to increase in the vehicle-treated RT group compared to the vehicle-treated control group (Figure 7A). However, the LV expressions of the pro-inflammatory *Il1*, interleukin-6 (*Il6*)*,* and tumor necrosis factor-α (*Tnf*) were significantly elevated in the sPIF-treated RT group compared to the vehicle-treated control group at week 1 (Figures 7A–C). Moreover, the LV expression of the pro-survival and anti-inflammatory (37) signal transducer and activator of transcription 3 (*Stat3*) tended to increase in the vehicle-treated RT and sPIF-treated RT groups at week 1 (Figure 7D). At week 3, the sPIF treatment markedly reduced the LV overexpression of *Il1* and *Il6* compared to the vehicle-treated control group (Figurers 7A,B). At week 3, the sPIF treatment significantly decreased the LV overexpression of *Stat3*, showing a similar expression pattern to *Il1* and *Il6* (Figure 7D). At week 15, there were no significant differences in the LV expressions of *Il1, Il6*, *Tnf,* and *Stat3* among the groups (Figures 7A–D).

**Figure 7:**
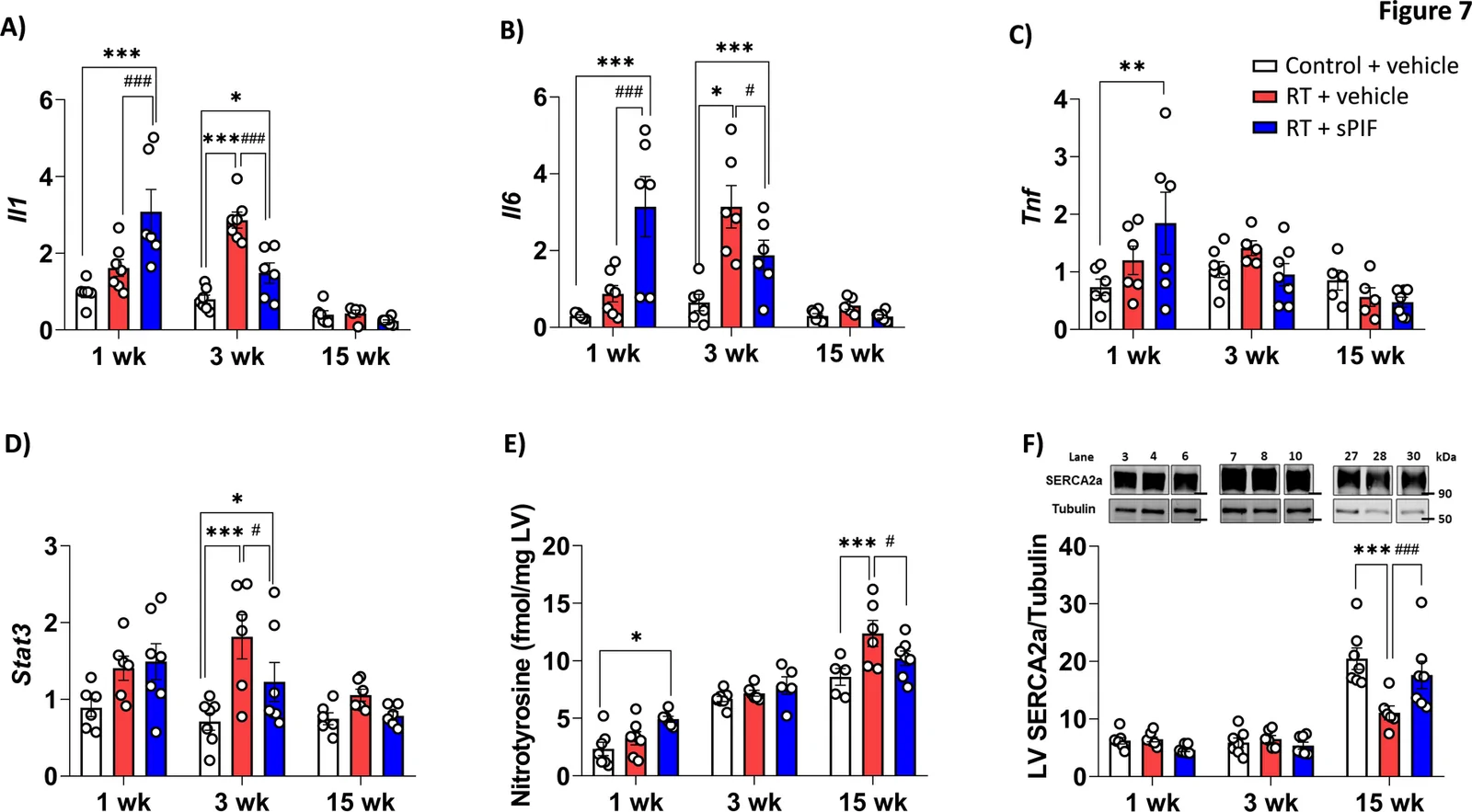
The effects of synthetic preimplantation factor (sPIF) on the molecular signs of inflammation, nitro-oxidative stress, and sarcoendoplasmic reticulum calcium ATPase 2a isoform (SERCA2a) in radiation-induced heart disease. **(A)** Left ventricular (LV) transcript levels of interleukin-1 (*Il1*), **(B)** interleukin-6 (*Il6*), **(C)** tumor necrosis factor-α (*Tnf*), **(D)** signal transducer and activator of transcription 3 (*Stat3*), **(E)** LV nitrotyrosine, and **(F)** LV protein levels of SERCA2a normalized to tubulin at weeks 1, 3, and 15. Values are presented as mean ± SEM, *p < 0.05, **p < 0.01, and ***p < 0.001 *vs.* vehicle-treated control, ^#^*p* < 0.05, and ^####^*p* < 0.001 *vs*. vehicle-treated RT, *n* = 6-7, two-way ANOVA, Holm-Sidak *post hoc* test. Western blot images were captured using the Odyssey CLx and exported with Image Studio 5.2.5. Black lines on the Western blot images show the grouping of the cropped blots. The full-length Western blots are presented in Supplementary Figures S17-21.

### 3.8 sPIF treatment reduced the nitro-oxidative stress in RIHD

After the first week, the overexpression of the inflammatory markers, *Il1, Il6,* and *Tnf,* was accompanied by a markedly higher LV level of nitrotyrosine, a marker of nitro-oxidative stress, in the sPIF-treated RT group compared to the vehicle-treated control group (Figure 7E). The GSH and GSSG levels were also increased in the irradiated groups compared to the vehicle-treated control group (Supplementary Figures 5A,B). Moreover, at week 1, the GSH level was markedly higher in response to sPIF treatment than in the vehicle-treated RT group (Supplementary Figure 5A). However, there was no significant difference in the GSH/GSSG ratios among the groups at week 1 (Supplementary Figure 5C). Riboflavin is the precursor of flavin adenine dinucleotide (FAD) and a cofactor of the antioxidant glutathione redox cycle, exerting cardioprotective effects by inhibiting oxidative stress and CM apoptosis (38, 39). At week 1, the LV riboflavin level was significantly reduced in the vehicle-treated RT group compared to the vehicle-treated control group (Supplementary Figure 5D). In response to sPIF, the riboflavin level was markedly higher compared to the vehicle-treated control or vehicle-treated RT groups (Supplementary Figure 5D). At weeks 3 and 15, there were no significant differences in the reduced and oxidized glutathione (GSH and GSSG, respectively) levels and the GSH/GSSG ratios among the groups (Supplementary Figures 5A–C). However, the LV riboflavin content remained markedly higher in the sPIF-treated RT group compared to the vehicle-treated control group (Supplementary Figure 5D). At week 15, significantly reduced nitrotyrosine levels were detected in the LVs after sPIF treatment compared to the vehicle-treated RT group, indicating its antioxidant properties (Figure 7E). Moreover, the nicotinamide adenine dinucleotide (NAD^+^) levels were markedly higher in both irradiated groups at week 1 as well as in the sPIF-treated RT group at week 15 compared to the time-matched vehicle-treated control groups (Supplementary Figure 5E).

### 3.9 sPIF treatment mitigated the LV repression of sarcoendoplasmic reticulum calcium ATPase 2a isoform at week 15

To further investigate the molecular aspects of the RT-induced diastolic dysfunction, LV sarco-endoplasmic reticulum calcium ATPase 2a isoform (SERCA2a) levels were measured by Western blot (Figure 7F). At weeks 1 and 3, there were no significant differences in the LV SERCA2a levels among the groups (Figure 7F). At week 15, the LV SERCA2a level was significantly reduced in the vehicle-treated RT group compared to the vehicle-treated control group (Figure 7F). Importantly, the sPIF treatment was associated with higher SERCA2a protein level compared to the vehicle-treated RT group at week 15 (Figure 7F).

### 3.10 sPIF modulated early and late apoptotic markers in a time-dependent manner

At week 1, the LV expression of the pro-apoptotic *Bax* was markedly increased in the sPIF-treated RT group compared to the vehicle-treated control group (Figure 8A). There was no significant difference in the LV expression of the anti-apoptotic *Bcl2* among the groups at week 1 (Figure 8B). At week 1, the *Bax/Bcl2* ratio tended to increase in the vehicle-treated RT group and increased markedly in the sPIF-treated RT group (Figure 8C). There was no significant difference in BAX and BCL2 expression at the protein level between the vehicle-treated RT and time-matched vehicle-treated control groups at any follow-up time point (Figures 8D,E). However, at week 1, the LV BAX level was significantly higher in the sPIF-treated RT group compared to the vehicle-treated control group (Figure 8D). At week 1, the BAX/BCL2 ratio tended to increase in the RT-only group compared to the vehicle-treated control group (Figure 8F). At week 3, *Bax* was overexpressed in both irradiated groups compared to the vehicle-treated control group (Figure 8A). In contrast, the LV expression of *Bcl2* showed an increasing tendency in the vehicle-treated RT group compared to the vehicle-treated control group (Figure 8B). At week 3, the *Bax/Bcl2* ratio at the transcript level as well as the BAX/BCL2 ratio at the protein level were significantly elevated in both irradiated groups compared to the vehicle-treated control group (Figures 8C,F). At week 15, LV transcript levels of *Bax* and the *Bax/Bcl2* ratio did not differ significantly among groups; however, *Bcl2* expression was markedly reduced in the sPIF-treated RT group compared with the vehicle-treated control group (Figures 8A–C).

**Figure 8:**
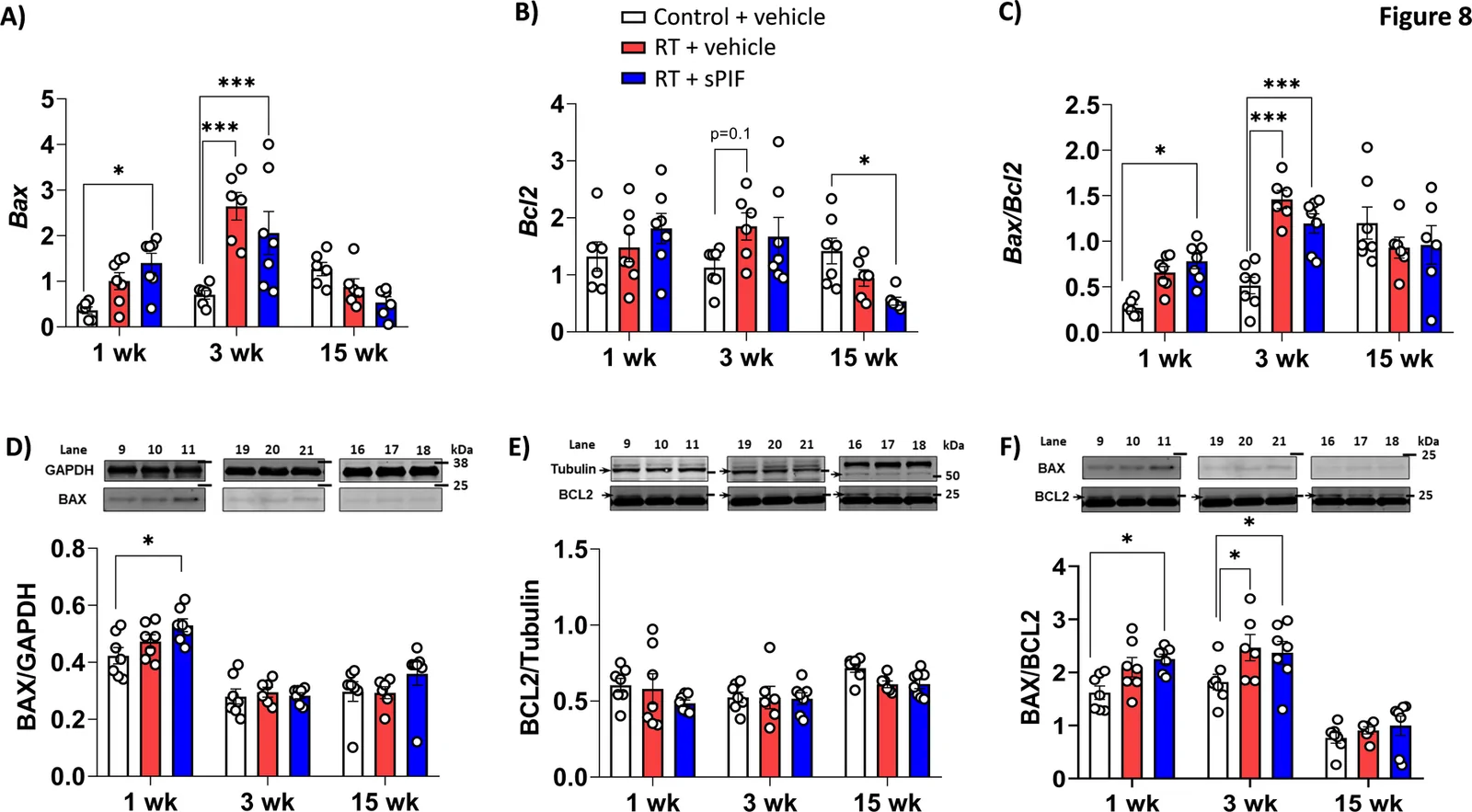
The effects of synthetic preimplantation factor (sPIF) on the molecular signs of apoptosis in radiation-induced heart disease. Left ventricular (LV) transcript levels of **(A)** the BCL2-associated X apoptosis regulator gene (*Bax*) and **(B)** BCL2 apoptosis regulator gene (*Bcl2*), **(C)** *Bax/Bcl2* ratio, LV protein levels of **(D)** BAX normalized to GAPDH, and **(E)** BCL2 normalized to tubulin, and **(F)** BAX/BCL2 ratio at weeks 1, 3, and 15. Values are presented as mean ± SEM, *p < 0.05, and ***p < 0.001 *vs*. vehicle-treated control; *n* = 6- 7; two-way ANOVA; Holm- Sidak *post hoc* test. Western blot images were captured using the Odyssey CLx and exported with Image Studio 5.2.5. The full-length Western blots are presented in Supplementary Figures S22-32.

The LV expression of later apoptotic markers, including caspases (*Casp*) and apoptosis-inducing factor (AIF), was measured at the endpoints (Supplementary Figures 6A–F). At week 1, there were no significant differences in the LV expression of caspases and AIF among the groups (Supplementary Figures 6A–F). At week 3, *Casp2*, *Casp3*, and *Casp7* were overexpressed in the RT-only group compared to the vehicle-treated control group (Supplementary Figures 6A–C). The sPIF treatment significantly reduced the overexpression of *Casp3* and *Casp7* compared to the vehicle-treated RT group at week 3 (Supplementary Figures 6B,C). At week 15, *Casp2* expression was significantly decreased in both irradiated groups (Supplementary Figure 6A). *Casp7* expression tended to decline in the RT-only group and was markedly reduced in the sPIF-treated RT group compared to the vehicle-treated control group (Supplementary Figure 6C).

### 3.11 sPIF treatment mitigated cellular hypertrophy and injury in H9c2 cardiomyoblasts

The H9c2 cardiomyoblasts’ cell viability was not affected by ANG II (1 µM), sPIF (in a concentration range of 50 ng/ mL to 5 *μ*g/mL), or their combination (Figure 9A). ANG II significantly increased the size of H9c2 cardiomyoblasts compared to the control group (Figure 9B). The size of the sPIF-treated (50 ng/mL) cardiomyoblasts was similar to that of the control group and was significantly smaller compared to the ANG II-treated group (Figure 9B). Interestingly, the cell size of the group treated with the combination of ANG II and sPIF was not significantly different from that of the ANG II-treated group or the control group (Figure 9B). The co-treatment with sPIF markedly counterbalanced the reduced viability of H9c2 cardiomyoblasts exposed to hydrogen peroxide (H_2_O_2_, Figure 9C).

**Figure 9:**
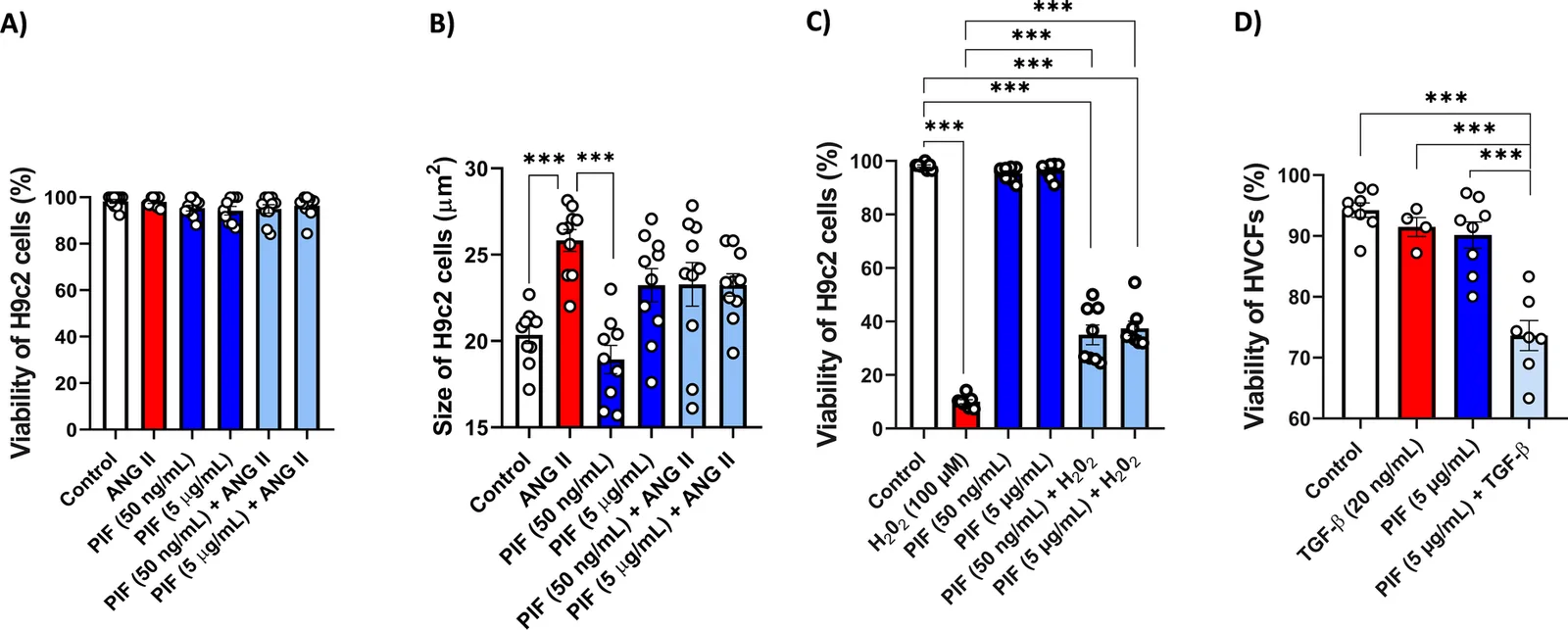
The effects of synthetic preImplantation factor (sPIF) on H9c2 cardiomyocytes treated with angiotensin II (ANG II) or hydrogen peroxide (H2O2) and human ventricular cardiac fibroblasts (HVCFs) treated with transforming growth factor-*β*1 (TGF-*β*1). **(A)** viability of H9c2 cells treated with ANG II, **(B)** size of H9c2 cells treated with ANG II, **(C)** viability of H9c2 cells treated with H_2_O_2_, and **(D)** viability of HVCFs treated with TGF-*β*1. Data are expressed as mean ± SEM, *n* = 6-10 replicates/group; *p < 0.05, **p < 0.01, and ***p < 0.01, one-way ANOVA, Holm-Sidak *post hoc* test.

### 3.12 sPIF treatment reduced the viability and collagen expression of HVCFs under TGF-*β*1-induced stress conditions

The viability of HVCFs was not influenced by TGF-*β*1 or sPIF alone (Figure 9D). Surprisingly, in the presence of TGF-*β*1, the higher concentration of sPIF reduced the cell viability of HVCFs compared to the control group (Figure 9D). Moreover, sPIF treatment significantly reduced collagen-I and *α*-smooth muscle actin (*α*SMA) protein expression in HVCFs after exposure to TGF-*β*1 (Supplementary Figures 7, 8, respectively).

### 3.13 sPIF treatment and cell migration in HVCFs

TGF-*β*1 can transiently enhance migration but ultimately delays migration and scratch closure in cardiac fibroblasts by driving differentiation into contractile *α*SMA^+^ myofibroblasts, strengthening focal adhesions, and promoting excessive ECM deposition with reduced remodeling. This ultimately creates a stiff, adhesive environment that suppresses motility and proliferation, particularly at later stages of development. In line with that, in our scratch assays, TGF-*β*1 significantly increased the motility of HVCFs within 1 h after scratching (Supplementary Figures 9A), consistent with its early pro-migratory activity. During the early phase (1–6 h), sPIF alone or combined with TGF-*β*1 further supported cell migration (Supplementary Figures 9A–C). However, in the later phases (24–30 h), TGF-*β*1 markedly delayed HVCF motility, as expected (Supplementary Figures 9D,E). Notably, at 30 h, sPIF alone and in combination with TGF-*β*1 appeared to support migration (Supplementary Figure 9E). These findings suggest that TGF-*β*1 exerts biphasic effects on fibroblast migration (i.e., initially promoting but later suppressing motility through fibrotic programming) while sPIF enhances migration in the early phase and may partially counterbalance late-stage inhibitory effects. These dynamics underscore the complexity of TGF-*β*1 signaling in cardiac fibroblasts, suggesting that sPIF modulation may have therapeutic relevance in pathological fibrosis.

### 3.14 sPIF treatment enhanced the anti-proliferative effects of RT in human breast cancer cell lines

The effects of sPIF on the proliferation of three different human breast carcinoma cell types (MCF-7, T47D, and MDA-MB-231) were investigated using a broad concentration range (0.5 ng/mL-15 μg/mL, Supplementary Figures 10A–C). None of the tested concentrations of sPIF increased cell proliferation (within 72 h) in the human breast cancer cell lines (Supplementary Figures 10A–C). Moreover, sPIF intensified the anti-proliferative effect of RT in breast cancer cell lines compared to RT alone (Supplementary Figures 10D–F).

## 4 Discussion

Here, we demonstrated that sPIF treatment mitigated RIHD progression by reducing LVH, diastolic dysfunction, cardiac fibrosis, and selected markers of nitro-oxidative stress during the chronic phase of RIHD (15 weeks post-RT) in an established rat model (32). Additionally, sPIF treatment preserved LV diastolic function and lowered inflammatory markers during the subacute phase of RHID (3 weeks post-irradiation), which presumably enabled fibrosis prevention at 15 weeks. The cardioprotective effects of sPIF were further validated *in vitro* using H9c2 cardiomyoblasts and HVCFs. sPIF even enhanced RT's inhibitory effects on breast cancer cell proliferation. These findings may suggest that sPIF may serve as a potential dual cardioprotective and anti-proliferative agent, warranting further mechanistic and clinical studies.

RT-induced diffuse myocardial injury is a progressive disease that develops over several years post-irradiation and depends primarily on the RT dose in humans (10, 12). It can lead to cardiomyopathy, cardiac fibrosis, and HF (9–11). In patients, RT-induced cardiac damage encompasses a spectrum of abnormalities, including diastolic dysfunction, LVH, and fibrosis, which may manifest as a complex form of HFpEF (7, 40, 41). Indeed, in our present study, rats with RIHD showed progressive LVH development and diastolic functional deterioration from week 3, with proportionally reduced LV volumes, SV, and CO, and preserved EF at week 15, in consonance with previous studies (31, 32, 40, 41). Interestingly, FS was significantly altered, whereas EF remained unchanged after RT at week 15. This apparent discrepancy may partly reflect differences in the methodology used to determine these parameters. FS represents the relative change in LV internal diameter between end-diastole and end-systole and is therefore a linear and geometry-dependent measure of LV systolic function (42). In the present study, FS was derived from LV dimensions obtained in the parasternal long-axis view, whereas EF was determined from four-chamber-view images based on LV volumetric measurements. In contrast to FS, EF reflects the relative change in LV chamber volume during systole and provides a more global estimate of LV systolic performance (42, 43). Therefore, alterations in LV dimensions and geometry associated with cardiac remodeling may influence FS without necessarily resulting in a proportional change in EF, which is also substantially influenced by loading conditions. Moreover, preserved EF does not exclude subtle alterations in myocardial mechanics (44). Thus, the observed alteration in FS in the presence of preserved EF may reflect changes in LV geometry and/or subtle systolic mechanics rather than overt impairment of global LV systolic function.

Because diastolic dysfunction and molecular, echocardiographic, or histological signs of LVH were not detectable at week 1 post-irradiation, we aimed to initiate preventive sPIF treatment before RT-induced cardiac changes began. We implemented this intervention in three distinct phases of RIHD development: acute/early (1-week follow-up), subacute (3-week follow-up), and chronic (15-week follow-up). This provided important insight into sPIF-induced protection in the acute injury, subacute inflammatory, and chronic fibrotic phases of RIHD. This approach was supported by the reported systemic and local anti-inflammatory, antioxidant, tissue-reparative, and radioprotective effects of sPIF (16, 24, 28, 33, 45, 46). Daily administration during the first 2 weeks was intended to provide early protection, whereas subsequent twice-weekly treatment allowed evaluation of sustained protection against progressive cardiac injury and fibrosis. The extended regimen was adopted from previous episodic sPIF administration (25), while clinical tolerability data support the feasibility of subcutaneous administration (34).

In our study, sPIF administration improved echocardiographic parameters, histological signs, and molecular markers (*Myh7*/*Myh6* ratio) of RT-induced LVH at week 15. Unexpectedly, body weight and the ratios of total heart and LV weights to tibia length were significantly lower in both RT groups at week 15. This phenomenon likely resulted from the high RT dose we used, which caused severe initial cell damage, as shown by increased BAX/BCL2 ratios at weeks 1 and 3 post-RT. Consequently, the surviving CMs presumably developed compensatory LVH, as confirmed in our study, to maintain cardiac pump function and ensure adequate oxygen supply despite the smaller heart and LV weights relative to tibia length. Furthermore, in the present study, the sPIF treatment ameliorated the severity of RT-induced compensatory LVH at week 15. Here, we found beneficial effects of sPIF on the RT-induced diastolic dysfunction based on IVRT, e’, and E/e’ results at week 15. Moreover, sPIF prevented a reduction in LV SERCA2a protein level compared with the RT group. Interestingly, more preclinical studies have found a significant inverse correlation between SERCA2a gene expression and IVRT, indicating impaired relaxation and a potential association with HFpEF (47, 48). Moreover, the antioxidant and anti-fibrotic properties of sPIF likely also contributed to the improvement of diastolic dysfunction and LVH in our RIHD model at week 15.

Increased cell death, nitro-oxidative stress, and inflammation have been confirmed to contribute to LVH and fibrosis in RIHD (7, 8, 14). Additionally, the chronic upregulation of IL6/STAT3 signaling has been reported to play a key role in the development of LVH and fibrosis in preclinical and clinical studies (49, 50). In line with that, several early and advanced molecular signs of apoptosis (i.e., increased BAX/BCL2 ratio at weeks 1 and 3, and overexpression of *Casp2*, *Casp3*, and *Casp7* at week 3), inflammatory cytokines (i.e., overexpression of *Il1 and Il6* at week 3), fibrotic and remodeling-associated markers (i.e., overexpression of *Stat3* and *Mmp2* at week 3) preceded the echocardiographic and histologic signs of LVH and fibrosis in the vehicle-treated RT group, similarly to previous findings in RIHD models (31, 32, 40, 41). Synthetic PIF treatment reduced LV overexpression of *Casp3* and *Casp7* at week 3 post-irradiation and preserved the viability of H9c2 cells exposed to hydrogen peroxide-induced oxidative stress*,* suggesting potential anti-apoptotic effects of sPIF, as reported in previous studies (21, 25, 26, 29). At week 15 post-irradiation, the mRNA levels of *Casp2, Casp7*, and *Casp9* were reduced in both irradiated groups, irrespective of sPIF treatment. However, protein expression levels of CASP7, AIF, BAX, and BCL2 did not differ significantly among the groups. In our present study, these inconsistencies in apoptosis data likely reflect biphasic effects of sPIF over time. Initially, an increased BAX/BCL2 ratio in response to sPIF might indicate activation of apoptosis to clear damaged cells, consistent with the overexpression of inflammatory markers (*Il1* and *Il6*) and increased nitrotyrosine levels observed at week 1. Later, reduced caspase expression at the transcript level and stable apoptotic markers at the protein level might suggest a shift toward cell survival and tissue repair. By week 15, sPIF appeared to prevent chronic cell death and limit harmful remodeling in RIHD. Indeed, this complements the anti-apoptosis effects observed in human trophoblast cells, developing embryos, and neurons through specific molecular pathways (15, 18, 21), including i) decreasing p53 phosphorylation, thereby preventing pro-apoptotic activation (21); ii) upregulating anti-apoptotic genes like BCL2 and downregulating pro-apoptotic proteins like BAX and BAK (21); iii) targeting proteins that protect against oxidative stress (PBI/TRX) and protein misfolding (HSPs) (28); iv) boosting neuroprotection via PKA/PKC signaling pathways (51); and v) regulating maternal natural killer (NK) cells and T-cells to create a favorable, controlled pro-inflammatory environment without causing systemic immune suppression (16, 52).

Beyond its anti-apoptotic effects, PIF acts as a targeted, pregnancy-derived immunomodulatory peptide that preserves homeostasis and basal immunity with a favorable immune shift rather than inducing a detrimental, broad-spectrum inflammatory response. By preserving antipathogen action, sPIF regulates global activated immunity and promotes immunoprotection. This synergizes with the affected organ and tissue type to promote integrated repair and restored function, thereby enhancing embryo acceptance, neuroprotection, radiation protection, and tissue repair (19, 22, 45, 53). sPIF was reported to alter the immune environment to favor tolerance and healing through the following mechanisms: i) regulating innate immunity by blocking the voltage-gated potassium (K_V_1.3) channels and thereby reducing overactivity of neutrophils and NK cells without broad immune depletion (53–55); ii) modulating cytokines by decreasing pro-inflammatory ones in autoimmune and maternal-fetal models, while balancing cytokine levels in immune cells (36, 56), iii) selectively tuning adaptive immunity by inhibiting pro-inflammatory pathways like IL-23 and IL-17a-mediated pathways, thereby reducing toxicity but preserving infection defense (17); and iv) supporting tissue and cellular repair, therefore showing therapeutic promise for autoimmune diseases and localized inflammation by aiding tissue remodeling and protecting the nervous system (33, 36).

In the context of ionizing radiation, sPIF has been studied as a medical countermeasure to mitigate radiation-induced damage and ARS (24). sPIF has been reported as a robust immunomodulator and cellular protector in ARS by targeting the biological cascades triggered by total-body or localized radiation, binding to innate immune cells (macrophages and neutrophils), suppressing inflammatory mediators such as TNF-α and iNOS, and promoting a tolerant, balanced immune profile in ARS. sPIF has also been shown to target key intracellular chaperones, PDI/TRX and HSPs, thereby directly reducing oxidative stress and protecting critical cellular structures (24). sPIF-induced protection against ARS was analyzed in the jejunum (24). Shortly after sublethal radiation, global genomic and pathway analyses revealed that sPIF induced specific protective effects that prevented progression to chronic inflammation. These protective effects of sPIF involved mitochondrial control, ROS reduction, regulation of Ca^2+^ flux, and reduced inflammation induced by Panx and Cxcl13 (24). In contrast, sPIF exerted anti-apoptotic and anti-infective effects and increased expression of lysozyme-1, olfactomedin-4, and CSP6-caspase (24). Importantly, sPIF also reduced oncogenic potential by modulating cyclin-dependent protein kinase (*Ccnd*) expression in the GI tract (24). Notably, sPIF was tested in Fast Track FDA human clinical trials for autoimmune disease, demonstrating safety and the absence of adverse drug-drug interactions (34). Further, sPIF safety was demonstrated in FDA-directed toxicology and toxicokinetic studies, in which daily high-dose sPIF administration for 6 months in mice and 9 months in dogs showed no observable adverse effects (NOAEL) (34). This supports progress towards clinical application since sPIF has also received FDA orphan drug designation for ARS based on studies conducted with NIH/NIAID. Interestingly, in our present study, sPIF also enhanced the effectiveness of RT in slowing the growth of human breast cancer cells. This is in line with sPIF-induced anti-neoplastic effects (17).

We also analyzed the putative oxidative and nitrosative signaling mediators in LV tissue following RT and sPIF treatment in our present study. Importantly, the glutathione redox cycle cofactor riboflavin was significantly lower in the RT group at week 1. However, there were no significant changes in the GSH/GSSG ratios across groups at any time point. The NAD^+^/NADH + H^+^ system also supports antioxidant mechanisms by increasing GSH levels and antioxidant enzyme activity (57, 58). Indeed, in our present study, NAD^+^ levels were elevated during the early phase of RIHD in both irradiated groups (i.e., week 1) and the sPIF-treated RT group 15 weeks post-irradiation. Supporting our results, increased NAD^+^ levels have been reported in acute inflammatory conditions and shown to improve mitochondrial respiration and attenuate inflammation in LVH and heart failure in preclinical and clinical studies (59, 60). We observed hereby antioxidant (i.e., increased riboflavin level at week 1 and decreased nitrotyrosine level at week 15, increased viability of H_2_O_2_ plus sPIF-treated H9c2 cardiomyoblasts) and anti-inflammatory (i.e., repression of *Il1* and *Il6* at week 3) effects of sPIF in concordance with its previously published effects in other animal models and clinical studies (17–19, 23, 24, 61). In contrast, one week after irradiation, sPIF treatment resulted in elevated nitrotyrosine levels and overexpression of *Tnf, Il1, and Il6* compared to the control group in our present study. We hypothesized that the enhanced acute inflammation induced by sPIF might facilitate the elimination of tissue debris and contribute to the earlier start of adaptive mechanisms (*e.g*., LVH development indicated by an elevated *Myh7/Myh6* ratio) and wound healing processes (fibrosis induction indicated by *Tgfb* overexpression) in this early phase of RIHD, mainly due to the immunomodulatory effects of sPIF as discussed above (16, 58, 62). In the present study, at week 15, the LV expression levels of the inflammatory markers *Il1*, *Il6*, and *Tnf* did not differ significantly among the groups in our hands. In our previous study using a similar RIHD model, *Il6* was overexpressed; however, *Il1* and *Tnf* expressions failed to change in response to RT at week 15 (32). This decline in inflammatory gene expression may suggest reduced inflammation at this chronic follow-up time point in our RIHD model, similar to the apoptosis markers.

The TGF-*β*1/SMAD2/3-mediated pathway is considered the key molecular mechanism in radiation-induced cardiac fibrosis (63, 64). Significantly decreased cell viability and collagen-I protein expression in TGF-*β*1-induced HVCFs were observed after administration of sPIF compared with the untreated condition. We next examined whether sPIF changed the migration capacity of HVCFs in the presence of TGF-*β*1. We observed a biphasic influence of TGF-*β*1 on cardiac fibroblast motility, consistent with prior reports describing its context- and time-dependent regulation of cell migration (65). In the early phase after scratching, TGF-*β*1 transiently enhanced HVCF migration, in line with evidence that low-level or acute TGF-*β*1 exposure can promote motility via SMAD-independent activation of MAPK and focal adhesion kinase or Rho GTPases (66, 67). However, at later phases, TGF-*β*1 markedly impaired migration, reflecting its canonical induction of myofibroblast differentiation, stress fiber assembly, and excessive ECM deposition, which collectively restrict motility and favor fibrotic remodeling (65). Notably, sPIF significantly supported migration in the early phase and abrogated TGF-*β*1's late inhibitory action. Its ability to sustain fibroblast motility may support a role in modulating fibroblast-driven repair while mitigating maladaptive fibrosis. Moreover, our results on the effects of sPIF alone and in coadministration with TGF-*β*1 on HVCF's cell motility at 1–6 h are in line with the sPIF effect on promoting trophoblast invasion (22). In addition, interstitial fibrosis and overexpression of *Ctgf* and SMAD2/3 in the left ventricles 15 weeks post-irradiation also highlighted the antifibrotic potential of sPIF. In consonance with our present findings, Morgoulis et al. demonstrated sPIF-mediated reduction of cardiac and other muscle fibrosis in a mouse model of Duchenne muscular dystrophy (27).

## 5 Limitations

The authors recognize several limitations of the current study. Significant differences exist in the pathomechanisms of experimental and clinical RIHD, comprising species-, sex-, and age-related attributes, as well as the absence of tumors, atherosclerosis, and other cardiovascular risk factors (68, 69). To avoid cycle- and hormone-dependent protective effects, we opted to use only male rats in the present study. Moreover, we tested the therapeutic effects of sPIF in a healthy adolescent rat model of RIHD, which represents an additional limitation. In our model, RT-induced diffuse myocardial injury, concomitant inflammation, and interstitial fibrosis were comparable to those observed clinically. However, we recognize that single-dose irradiation models differ from clinical radiotherapy, which typically uses multiple smaller fractions. Single RT doses can exaggerate and accelerate tissue damage in preclinical models. Single RT doses can alter biological responses, such as inflammation and fibrosis, and may not fully reflect the chronic, cumulative effects seen in patients. Consequently, findings from single-dose models might have limited translational relevance. In our present study, with the aims of multimodal approximation and the investigation of all three phases of radiation injury (acute inflammation, subacute tissue reorganization, and fibrosis), we used a single fraction of a very high biological RT dose with drastic consequences (30–32). The selected dose was based on previous dose-finding experiments (10–50 Gy localized to the heart), resulting in measurable alterations, particularly fibrosis development assessed by histology, that could be modified by the interventions used, and the animals could still be maintained alive until the end of the planned experiment duration (30–32). Notably, in modern RT practice, very high RT doses are used in stereotactic radiosurgery; our findings may also apply to that field (70). Moreover, sPIF had previously been tested and demonstrated 100% protection against lethal radiation; thus, its use in that model was relevant (24). Similar high RT doses and experimental endpoints were used by others (71, 72). Animal models incorporating both sexes, mediastinal tumors or breast cancer, cardiovascular risk factors, comorbidities, and advanced age would more closely recapitulate the clinical setting of radiation-induced heart disease (RIHD). Developing and characterizing such models represents an important direction for future preclinical research. In the present study, RT-qPCR was used as a broad screening approach to characterize the transcriptional regulation of several apoptosis-related genes, including *Bax, Bcl2, Casp2, Casp3, Casp7*, and *Casp9*. In contrast, Western blot analysis was performed on a selected panel of apoptosis-related proteins (BAX, BCL2, CASP7, and AIF) rather than all targets investigated at the mRNA level. Caspase-7 was selected as a representative executioner caspase for protein-level assessment (73). In parallel, BAX and BCL2 were assessed as key regulators of mitochondrial apoptotic signaling, while AIF was included to provide information on the caspase-independent mitochondrial cell death pathway (74, 75). Thus, the Western blot experiments were intended to complement the broader transcriptional analysis by examining selected components of both caspase-dependent and caspase-independent apoptotic pathways rather than to provide protein-level validation of every caspase analyzed by RT-qPCR. We are aware that protein-level assessment of CASP2, CASP3, and CASP9, particularly their cleaved/activated forms, would provide a more comprehensive characterization of apoptotic signaling. We therefore acknowledge the absence of these measurements as a limitation of the present study. Moreover, Western blotting is a semi-quantitative method for assessing protein expression and can pose many challenges, including the proper selection of a loading control (76). Therefore, further methods are needed to verify the protein expression changes assessed by Western blot. We acknowledge that H9c2 cells retain an immature cardiomyoblast-like phenotype and do not completely reproduce the characteristics of differentiated adult rat cardiomyocytes. Nevertheless, given the extensive validation of Ang II-induced hypertrophic responses in H9c2 cells and the complementary use of HVCFs to assess collagen expression, we believe the selected models were appropriate for addressing the specific experimental questions of the present study. We agree that future validation in human cardiomyocyte-derived models, such as AC16 cells or hiPSC-derived cardiomyocytes, would further strengthen the translational relevance of our findings.

## 6 Conclusions

RIHD is a common complication of RT for thoracic tumors and breast cancer, often leading to heart failure characterized by diastolic dysfunction, LVH, and cardiac fibrosis. Key contributors to RIHD include nitro-oxidative stress, inflammation, fibrosis, and cell death. Here, we showed that systemic administration of sPIF for 14 days, followed by twice-weekly maintenance doses, mitigated RIHD progression in rats by time-dependently ameliorating nitro-oxidative stress and inflammatory markers, as well as echocardiographic and histologic signs of diastolic dysfunction, LVH, and fibrosis. However, our results show clear changes in several nitro-oxidative stress, inflammatory, and fibrosis markers; the exact initiating targets and upstream signaling mechanisms of sPIF in RIHD remain unexplored. Therefore, TGF-*β*/SMAD signaling and SERCA2a preservation are correlative biomarkers rather than definitive proof of direct mechanisms. Future preclinical studies could clarify the cardioprotective and anti-neoplastic mechanisms of sPIF and its direct mechanistic links to nitro-oxidative stress, inflammation, fibrosis, and apoptosis in RIHD. Our study identified sPIF as a novel, promising, druggable candidate for preventing and improving the signs of RIHD. Given its effectiveness in preclinical models, sPIF could be a valuable addition to cancer RT regimens and warrants further exploration of its safety and efficacy in human trials.

## Data Availability

The original contributions presented in the study are included in the article/Supplementary Material, further inquiries can be directed to the corresponding authors.
