# Supplementary material for: Synthetic preimplantation factor ameliorates the severity of radiotherapy-induced heart disease in a rat model
Source: Front Cardiovasc Med. 2026 Oct 16;13:1946843. doi: 10.3389/fcvm.2026.1946843 (PMC13719410; doi:10.3389/fcvm.2026.1946843)
Supplement: Supplementary file 1 [file Table1.docx]

**Supplementary Material**

**1. Supplementary methods**

1.1. Transthoracic echocardiography (TTE)

Cardiac morphology and function were assessed by TTE as described previously (1, 2) at weeks 1, 3, and 15 to monitor the development of radiation-induced heart disease (RIHD). Rats were anesthetized with 2% isoflurane (Forane, Aesica, Queenborough Limited, Queenborough, UK). Two-dimensional B-mode, M-mode, Doppler, tissue Doppler, and four-chamber-view images were performed by the criteria of the American Society of Echocardiography with a Vivid IQ ultrasound system (General Electric Medical Systems, New York, NY, USA) using a phased array 5.0–11 MHz transducer (General Electric 12S-RS probe, General Electric Medical Systems, New York, NY, USA). Data from three consecutive heart cycles were analyzed (EchoPac Dimension v201, General Electric Medical Systems, USA) by two experienced investigators in a blinded manner. The mean values of three measurements were calculated and used for statistical evaluation. Systolic and diastolic wall thicknesses were obtained from the parasternal short-axis view at the level of the papillary muscles (in the cases of the anterior wall) and the long-axis view at the level of the mitral valve (in cases of septal and posterior walls). The left ventricular internal diameters at the end of systole and diastole (LVESD and LVEDD) were measured by M-mode echocardiography from the long-axis view between the endocardial borders. The relative wall thickness was calculated using the formula (2xPWTd)/LVEDD. The fractional shortening (FS) was measured on M-mode images in the long-axis view. FS was used as a measure of cardiac contractility (FS = [LVEDD − LVESD]/LVEDD × 100). Diastolic function was assessed using pulsed-wave Doppler across the flow through the mitral valve and tissue Doppler imaging on the septal and lateral sides of the mitral annulus from the apical four-chamber view. Early (E) mitral inflow and septal, lateral, and their mean mitral annulus velocity (e') were used as an indicator of the diastolic function. Heart rate was calculated using pulsed-wave Doppler images. At the same time, transvalvular flow velocity profiles were measured according to the length of three consecutive heart cycles (measured between the start points of the E waves). Isovolumic contraction time (IVCT), ejection time (ET), and isovolumic relaxation time (IVRT) were measured using pulsed-wave Doppler across the flow through the mitral valve. IVCT was measured as the time from mitral valve closure to aortic valve opening. ET was determined as the time from aortic valve opening to aortic valve closure. IVRT was defined as the time from aortic valve closure to mitral valve opening. The myocardial performance (Tei) index was calculated using the formula (IVCT + IVRT) / ET. The left ventricular end-diastolic volume (LVEDV) and left ventricular end-systolic volume (LVESV) were calculated on four-chamber view images delineating the endocardial borders in diastole and systole. The stroke volume (SV) was calculated as the difference between LVEDV and LVESV. Ejection fraction (EF) was calculated using the formula (SV/LVEDV) × 100. Cardiac output (CO) was calculated as the product of SV and heart rate (HR).

1.2. Histologic analyses

Formalin-fixed paraffin-embedded subvalvular areas of the left ventricles were cut transversally into 5-μm sections and stained with hematoxylin-eosin (H&E) or picrosirius red/fast green (PSFG) stainings, as described previously (2, 3). Histological slides were scanned with a Pannoramic Midi II scanner (3D-Histech, Budapest, Hungary). Digital slide processing was performed in SlideViewer version 2.6 (last accessed on August 14, 2024). On the digital H&E images, CM perimeters and cross-sectional areas were measured to verify the development of LVH at the cellular level. Representative HE- and PSFG-stained slides were captured in Panoramic Viewer 1.15.4 (3D-Histech, Budapest, Hungary; https://old.3dhistech.com/pannoramic_viewer).

The Biology Image Analysis Software (BIAS 1.0, Single-Cell Technologies Ltd, Szeged, Hungary, https://single-cell-technologies.com/bias/) was used to evaluate HE images (2, 3). Image pre-processing was followed by deep learning-based cytoplasm segmentation. User-selected objects were forwarded to the feature extraction module, which is configurable to extract properties from the selected cell components. Here, transverse transnuclear cardiomyocyte (CM) perimeters and cross-sectional areas were measured in 100 consecutive mononucleated CMs selected based on longitudinal orientation and from a single cut surface (digitalized histological slide) of the left ventricular tissue blocks.

Cardiac fibrosis was assessed on PSFG slides with an in-house developed program as described previously (2–4). Briefly, this program determines the proportion of red pixels in the left ventricular (LV) sections using 2 simple color filters. For each red-green-blue (RGB) pixel, the program calculates the color of the pixel in the hue-saturation-luminance color space. The first filter is used to detect red portions of the image. The second filter excludes any white (empty) or light gray (residual dirt on the slide) pixel from further processing using a simple RGB threshold. In this way, the program groups each pixel into 2 sets: pixels considered red, and pixels considered green but not red, white, or grey. Red pixels in the first set correspond with connective tissue and fibrosis. Green pixels in the second set correspond to the cardiac muscle. Dividing the number of elements in the first set by the number of elements in both sets gives the proportion of the connective tissue compartment of the heart area examined.

1.3. Nitro-oxidative stress and redox status markers

LV samples were prepared, and nitrotyrosine, nicotinamide adenine dinucleotide (NAD^+^), reduced and oxidized glutathione (GSH and GSSG, respectively), and riboflavin were measured according to previously published methodologies using ultra-high performance liquid chromatography-tandem mass spectrometry (UHPLC-MS/MS) (5, 6). Multiple reaction monitoring  (MRM) transition of nitrotyrosine was 227.1/181.1 using 50 V as declustering potential and 16 V as collision energy, retention time: 6.5 min. MRM transition of riboflavin was 377.2/243.0 using 50 V as declustering potential and 28 V as collision energy, retention time: 10.1 min. MRM transition of NAD^+^ was 664.2/136.1 using 85 V as declustering potential and 50 V as collision energy, retention time: 2.0 min. MRM transition of GSH was 308.2/179.1 using 50 V as declustering potential and 14 V as collision energy, retention time: 1.8 min. MRM transition of GSSG was 614.4/485.1 using 50 V as declustering potential and 17 V as collision energy, retention time: 2.6 min. The extraction solvent of the heart tissues was 85% acetonitrile, and the ratio of extraction solvent to tissue was 22.2:1.

1.5. Transcription profiling by quantitative reverse transcription polymerase chain reaction (qRT-PCR) in left ventricular tissue samples

Quantitative RT-PCR was performed with gene-specific primers to monitor mRNA expression as described previously (2, 4). In the case of LV tissue, RNA was isolated using Qiagen RNeasy Fibrous Tissue Mini Kit (Qiagen, Hilden, Germany) and quantified by NanoDrop One Microvolume UV-VIS spectrophotometer (Thermo Fisher Scientific Inc., Waltham, MA, USA). Then, 100 μg of total RNA was reverse transcribed using iScript™ cDNA Synthesis Kit (BioRad Laboratories Inc., Hercules, CA, USA). The cDNAs were analyzed in technical duplicates using 10 μL reaction volumes. Specific primers and SsoAdvanced™ Universal SYBR® Green Supermix (BioRad Laboratories Inc., Hercules, CA, USA) were applied according to the manufacturer’s instructions using a BioRad CFX-96 machine with the accompanying BioRad CFX Manager (BioRad Laboratories Inc., Hercules, CA, USA) software for cycle threshold value analysis. Gene expression was calculated using the standard curve method (7). Ribosomal protein lateral stalk subunit P2 (*Rplp2*) and hypoxanthine-guanine phosphoribosyltransferase 1 (*Hprt1*) were used as control genes for normalization (1). The primers are listed in Supplementary Table 1.

1.6. Western Blot

To investigate the LV gene expression changes at the protein level, a standard Western blot technique was used as described previously (2, 4, 8). BAX (20 kDa), and SMAD2/3 (52 and 60 kDa) with GAPDH (37 kDa) loading control as well as apoptosis-inducing factor (AIF, 67 kDa), BCL2 (26 kDa), Caspase-7 (CASP7, 20 and 35 kDA), and SERCA2a (114 and 140 kDa) with tubulin (52 kDa) loading control were assessed at weeks 1, 3, and 15. LV samples (n = 6–7 in each group) were homogenized with an ultrasonicator (UP100H, Hielscher, Germany) in Radio-Immunoprecipitation Assay (RIPA) buffer (50 mM Tris-HCl (pH 8.0), 150 mM NaCl, 0.5% sodium deoxycholate, 5 mM ethylenediamine tetra-acetic acid (EDTA), 0.1% sodium dodecyl sulfate, 1% NP-40; Cell Signaling Technology Inc., Danvers, MA, USA) supplemented with phenylmethanesulfonyl fluoride (PMSF; Sigma-Aldrich, St. Louis, MO, USA) and sodium fluoride (NaF; Sigma-Aldrich, Saint Louis, MO, USA) to inhibit proteases and phosphatases, respectively. The crude homogenates were centrifuged at 15,000 × g for 30 min at 4 °C. After quantifying the supernatants’ protein concentrations using the BCA Protein Assay Kit (Pierce Thermo Fisher Scientific Inc., Waltham, MA, USA), 25 μg of reduced and denaturized protein was loaded. Then sodium dodecyl-sulfate polyacrylamide gel electrophoresis (SDS-PAGE, 10% gel, 50 V, 4 h) was performed followed by the transfer of proteins onto a nitrocellulose membrane (10% methanol in case of SMAD2/3, 20% methanol in the cases of AIF, BCL2, CASP7, SERCA2a, and tubulin and 15% methanol (SDS-free) in the cases of BAX and GAPDH, 35 V, 90 min). The efficacy of transfer was checked using Ponceau staining. The membranes were cut horizontally into parts corresponding to the molecular weights of BAX, SMAD2/3, GAPDH, AIF, BCL2, CASP7, SERCA2a, and tubulin. Membranes were blocked in 5% (w/v) bovine serum albumin (BSA, Sigma-Aldrich, Saint Louis, MO, USA) for 1 h and were incubated with primary antibodies diluted in 5% BSA in the concentrations of 1:1000 against AIF (#4642S), BAX (#14796), BCL2 (#ab196495), CASP7 (#9492S), SMAD2/3 (#8685T), SERCA2a (#9580), tubulin (#2144) or 1:5000 against GAPDH (#2118) overnight at 4 °C (BCL2 antibody was purchased from Abcam PLC, Cambridge, UK, and the others from Cell Signaling Technologies, Danvers, MA, USA).

Then, the membranes were incubated with IRDye® 800CW Goat Anti-Rabbit secondary antibody (LI-COR Biosciences, Lincoln, NE, USA) at a concentration of 1:5000 for 1 h at room temperature in 5% BSA to detect proteins of similar molecular weight on the same membrane, where applicable. Fluorescent signals were detected by the Odyssey CLx machine (LI-COR Biosciences, Lincoln, NE, USA), and digital images were analyzed and evaluated by densitometry with Quantity One Software (Bio-Rad Laboratories Inc., Hercules, CA, USA). Fluorescent signals were quantified using identical background-corrected regions of interest for each lane and normalized to (loading control/total protein). Full-length, unmodified Ponceau-stained membranes and cropped Western blot images are presented in the Supplementary Material (Figures S12-32). A more detailed description of the Western blot method used is provided in the Supplementary Material of Kovacs ZZA et al. (8).

1.7. Protective effects of sPIF against ANG II- and TGF-β1-induced stimuli in human ventricular fibroblasts (HVCFs)

HVCFs (Lonza, Switzerland) at passage 7-11 were seeded using a cell density of 1×10^5^ cells/mL. The cells were serum-starved for 24 hours and subsequently treated during the next 24 hours as follows: i) without treatment (control); ii) 1 µM angiotensin II (ANG II) (Sigma Aldrich, A9525); iii) 20 ng/mL transforming growth factor-β1 (TGF-β1, R&D Systems, 240-B); iv-v) ANG II + sPIF (50 ng/mL or 5 μg/mL); vi-vii) TGF-β1 + sPIF (50 ng/mL or 5 μg/mL) and viii-ix) sPIF (50 ng/mL or 5 μg/mL). The groups that received sPIF were pre-treated for 1 hour prior to ANG II or TGF-β1 exposure. The total RNA was extracted using the RNeasy Mini Kit (Qiagen) and reverse transcribed to cDNA using the QuantiTect reverse transcription kit (Qiagen). Samples were analyzed in technical duplicates using a CFX-Opus96 thermocycler with the accompanying CFX Manager software (Bio-Rad Laboratories Inc., USA). Specific human primers (Supplementary Table 1) were applied, and *Hprt1* served as a housekeeping gene for normalization. Relative gene expression was calculated by the 2^−ΔΔCt^ method.

To assess the migratory capacity of cells, the wound healing assay was performed according to the same protocol as described above for conditions i, iii, vi-vii, and viii-ix for 48 hours. An open area was created in the cell monolayer using a 200 µL tip. Images at baseline were taken immediately after creating the open wound area. Cells were imaged after migration to the scratched area 1, 3, 6, 24, and 30 hours using the Olympus Contrast Phase and the NIS Elements D Software system. For each time point, relative wound closure was calculated using Fiji software (n=6 replicates/condition). Relative „wound closure” was calculated by means of the change in scratch width over time, normalized to open area at baseline immediately after initiating the wound by cell scratching.

The cells were detached using trypsinization and incubated for 2 minutes with an equal volume of trypan blue (Gibco, 15250061). Cell viability and the cell concentration were determined using the Countess 3 Automated Cell Counter (Thermo Fisher). For immunocytochemical staining, 24 hours after seeding, the cells were treated with sPIF (50 ng/mL) for 1 hour, followed by co-exposure to TGF-ꞵ1 (20 ng/mL) for the next 24 hours. Afterwards, the cells were fixed in 4% paraformaldehyde diluted in PBS, and the permeabilization was performed using 0.1% Triton X-100. The samples were incubated for 1 hour at room temperature with the following primary antibodies: rabbit polyclonal anti-collagen-Iα1 (NovusBiologicals, 30054, 1:200) and mouse monoclonal anti-actin, Cy3 conjugated α-smooth muscle actin (αSMA, Sigma, C6198, 1:200). For collagen-Iα1, goat anti-rabbit IgG antibody (H+L) conjugated to Texas Red (Abcam, TI-1000, 1:200) was used. Coverslips were mounted to glass slides using a VECTASHIELD Antifade Mounting Medium with DAPI (H-1200-10). All immunocytochemical stainings were performed in triplicate. Stained cells were scanned using a laser scanning confocal microscope (LSM 700, Zeiss), and images were visualized using the ZEN software (Zeiss).

1.8. Protective effects of sPIF against oxidative stress in H9c2 cardiomyoblasts

H9c2 cells (American Type Culture Collection, ATCC, USA) at passage 18 were seeded into a 24-well plate at a density of 80×10^4^ cells/mL. After 24 hours, following the seeding, cell viability assays were performed. The cells were pre-treated with two different concentrations of sPIF (5 µg/mL and 50 ng/mL) for 1 hour and subsequently exposed to 100 µM hydrogen peroxide (H_2_O_2_, Honeywell, 216763) for 30 minutes. After exposure, the cells were washed with DPBS (Gibco, 14190144), and the medium was then changed to either standard culture medium or standard culture medium supplemented with sPIF, depending on the treatment group. Twenty-four hours following treatment, cell viability and cytotoxicity were assessed using XTT and LDH assays, as well as Trypan blue staining. The cytotoxicity was measured using the LDH-Cytox^TM^ Assay kit (BioLegend, 426401) following the manufacturer’s instructions. The Trypan blue staining was performed according to the above-described protocol. In the XTT viability assay, 1 mg/mL of XTT sodium salt (ChemCruz, sc-258336) with 5% phenazine methosulfate (Sigma-Aldrich, P9625) was added to the cells in an amount of 20% of the total culture medium volume and incubated for 4 hours at 37ºC. The absorbance was measured at the wavelength of 450 nm referenced to 630 nm on a multimode microplate reader (Spark, Tecan).

For the cell size assessment, 24 hours after seeding, the cells were pre-treated with two different concentrations of sPIF (5 µg/mL and 50 ng/mL) for 1 hour and then incubated with 1 µM Angiotensin II (Sigma Aldrich, A9525) for an additional 24 hours. Following the treatment, the trypan blue staining procedure was performed as described above. The samples were measured using the Countess 3 Automated Cell Counter (Thermo Fisher) to determine the average cell size. For all the tests, 10 replicates per group were used.

1.9. Proliferation assessment in human breast cancer cell lines

The effect of sPIF on breast cancer cell proliferation was determined on a panel of human adherent breast cancer cell lines. Human MCF-7 adenocarcinoma cell line with estrogen (ER), progesterone (PR), and glucocorticoid receptors, MDA-MB-231 differentiated triple negative adenocarcinoma cell line, and T47D luminal A subtype ER^+^, PR^±^, HER2^-^ cell line were purchased from ECCAC, Salisbury, UK. Cells were cultivated in a minimal essential medium (MEM) supplemented with 10% fetal bovine serum, 1% non-essential amino acids, and 1% antibiotic–antimycotic mixture (all from Capricorn Scientific Ltd., Ebsdorfergrund, Germany). The antiproliferative activity of sPIF (0.5 ng/mL – 15,000 ng/mL) on breast cancer cells was determined using a spectrophotometric MTT assay after a 72-hour treatment (9). Untreated samples served as controls. The cells’ viability was calculated and expressed as a percentage of absorbance values of treated and untreated cells.

The influence of sPIF on RT was also determined by using a modified MTT assay. 500 cells per well from each previously used cancer cell line were seeded and exposed to 4 Gy ionizing radiation and treated with sPIF (0.5 ng/mL to 15,000 ng/mL). Cells were incubated for 7 days, then living cells were assayed, and the inhibitory effect of RT combined with sPIF on cancer cell proliferation was evaluated. All cell-based experiments were carried out at least three times with five parallel repeats. A stock solution of sPIF (80 mg/mL) was prepared in deoxygenated phosphate-buffered saline.

# Supplementary Table 1

| Gene symbol | Gene name | Species | BioRad catalog No. /  Primer sequences |
| --- | --- | --- | --- |
| *Bax* | BCL2-associated X apoptosis regulator | rattus norvegicus | qRnoCED0002625 |
| *Bcl2* | B-Cell CLL/lymphoma 2 apoptosis regulator | rattus norvegicus | qRnoCED0006419 |
| *Casp3* | Caspase 3 | rattus norvegicus | qRnoCID0006430 |
| *Casp9* | Caspase 9 | rattus norvegicus | qRnoCID0006496 |
| *Col1* | Collagen type 1 alpha 1 chain | rattus norvegicus | qRnoCED0007857 |
| *Ctgf* | Connective tissue growth factor | rattus norvegicus | qRnoCED0001593 |
| *Hprt1* | Hypoxanthine-guanine phosphoribosyltransferase | rattus norvegicus | qRnoCED0057020 |
| *Il1* | Interleukin-1 | rattus norvegicus | qRnoCID0002056 |
| *Il6* | Interleukin-6 | rattus norvegicus | qRnoCID0053166 |
| *Mmp2* | Matrix metalloproteinase 2 | rattus norvegicus | qRnoCID0002887 |
| *Myh6* | α-myosin heavy chain | rattus norvegicus | qRnoCID0001766 |
| *Myh7* | β-myosin heavy chain | rattus norvegicus | qRnoCED0001215 |
| *Nppa* | A-type natriuretic peptide | rattus norvegicus | qRnoCED0006216 |
| *Rplp2* | Ribosomal protein lateral stalk subunit P2 | rattus norvegicus | forward: AGCGCCAAAGACATCAAGAA  reverse:  TCAGCTCACTGATGACCTTGTT |
| *Stat3* | Signal transducer and activator of transcription 3 | rattus norvegicus | qRnoCID0006149 |
| *Tgfb* | Transforming growth factor-β1 | rattus norvegicus | qRnoCID0009191 |
| *Tnf* | Tumor necrosis factor-α | rattus norvegicus | qRnoCED0009117 |
| *Acta* | α-smooth muscle actin | homo sapiens | forward:  GCAGCTCCAGCTATGTGTGAAG  reverse:  TTGTCCCATTCCCACCATCACC |
| *Col1* | Collagen type 1 alpha 1 chain | homo sapiens | forward:  AGTCGAGGGCCAAGACGAAG  reverse:  ACAACACCTTGCCGTTGTCG |
| *Col3* | Collagen type 3 alpha 1 chain | homo sapiens | forward:  GTGGACGTTGGCCCTGTTTG  reverse:  TGTCGGTCACTTGCACTGGT |
| *Gapdh* | Glyceraldehyde 3-phosphate dehydrogenase | homo sapiens | forward: TCCTGTTCGACAGTCAGCCG  reverse: CCCCATGGTGTCTGAGCGAT |
| *Tnf* | Tumor necrosis factor-α | homo sapiens | forward: TCCCCAGGGACCTCTCTCTA  reverse: GGGTTTGCTACAACATGGGC |

# Supplementary Table 2

# Supplementary Table 3

Supplementary Figure 1. The effects of synthetic preimplantation factor (sPIF) on the echocardiographic parameters related to left ventricular morphology during the development of radiation-induced heart disease. A) posterior wall thickness in diastole (PWTd), B) posterior wall thickness in systole (PWTs), C) septal wall thickness in systole (SWTs), D) anterior wall thickness in diastole (AWTd), and E) anterior wall thickness in systole (AWTs) at weeks 1, 3, and 15. Values are presented as mean ± SEM, *p<0.05, **p<0.01, and ***p<0.001 *vs*. vehicle-treated control, ^#^p<0.05, ^##^p<0.01, and ^##^p<0.01 vs. vehicle-treated RT, n=7-8, two-way ANOVA, Holm-Sidak *post hoc* test.

Supplementary Figure 2. The effects of synthetic preimplantation factor (sPIF) on LVESD, FS, and echocardiographic functional parameters related to relative wall thickness during the development of radiation-induced heart disease. A) Left ventricular end-systolic diameter (LVESD), B) fractional shortening (FS) obtained from the long-axis view, C) ratio of the half left ventricular end-diastolic diameter (LVEDD) to the posterior wall thickness in diastole (PWTd), and D) alternative formula for relative wall thickness calculated as (septal wall thickness in diastole [SWTd] + posterior wall thickness in diastole [PWTd])/left ventricular end-diastolic diameter [LVEDD]) at weeks 1, 3, and 15. Values are presented as mean ± SEM, ^**^p<0.01, and ^***^p<0.001 vs. vehicle-treated control, ^###^p<0.001 vs. vehicle-treated RT, n=7-8, two-way ANOVA, Holm-Sidak post hoc test.

Supplementary Figure 3. The effects of synthetic preimplantation factor (sPIF) on the echocardiographic parameters related to diastolic function during the development of radiation-induced heart disease. A) E velocity, B) septal mitral annulus velocity (e'), C) E / septal e’ ratio, D) lateral mitral annulus velocity (e'), and E) E / lateral e’ ratio at weeks 1, 3, and 15. Values are presented as mean ± SEM, ^**^p<0.01, and ^***^p<0.001 vs. vehicle-treated control, ^###^p<0.001 vs. vehicle-treated RT, n=7-8, two-way ANOVA, Holm-Sidak post hoc test.

Supplementary Figure 4. The effects of synthetic preimplantation factor (sPIF) on the echocardiographic parameters related to diastolic function and Tei index during the development of radiation-induced heart disease. A) isovolumic contraction time (IVCT), B) ejection time (ET), and C) myocardial performance (Tei) index calculated as (IVCT+IVRT)/ET at weeks 1, 3, and 15. Values are presented as mean ± SEM, ***p<0.001 vs. vehicle-treated control, ^###^p<0.001 vs. vehicle-treated RT, n=7-8, two-way ANOVA, Holm-Sidak post hoc test.

Supplementary Figure 5. The effects of synthetic preimplantation factor (sPIF) on oxidative stress markers in radiation-induced heart disease. Left ventricular (LV) A) reduced glutathione (GSH) levels, B) oxidized glutathione (GSSG) levels, C) reduced to oxidized glutathione ratio (GSH/GSSG), D) riboflavin, and E) nicotinamide adenine dinucleotide (NAD^+^) levels at weeks 1, 3, and 15. Values are presented as mean ± SEM, ^*^p<0.05, and ^**^p<0.01 vs. control, ^***^p<0.001 vs. vehicle-treated control, ^#^p<0.05, and ^###^p<0.001 vs. vehicle-treated RT, n=6-7, two-way ANOVA, Holm-Sidak post hoc test.

# Supplementary Figure 6. The effects of synthetic preimplantation factor (sPIF) on the molecular signs of apoptosis in radiation-induced heart disease. A) Left ventricular (LV) transcript levels of caspase-2 (*Casp2*), B) caspase-3 (*Casp3*), C) caspase-7 (*Casp7*), D) caspase-9 (*Casp9*), E) LV protein levels of caspase-7 (CASP7), and F) apoptosis-inducing factor (AIF) normalized to tubulin at weeks 1, 3, and 15. Values are presented as mean ± SEM, ^*^p<0.05, ^**^p<0.01, and ^***^p<0.001 *vs.* vehicle-treated control, ^#^p<0.05 *vs*. vehicle-treated RT, n=6-7, two-way ANOVA, Holm-Sidak post hoc test. Images were captured using the Odyssey CLx and exported with Image Studio 5.2.5. The full-length, unmodified Ponceau-stained membranes and cropped Western blots are presented in Supplementary Figures S28-32.

# Supplementary Figure 7. sPIF modulates collagen-I expression in human cardiac fibroblasts following TGF-β1 exposure. Human cardiac fibroblasts were pre-treated with sPIF (50 ng/mL) for 1 hour (Columns 2 and 4), followed by the addition of TGF-β1 (20 ng/mL) in the respective conditions (Columns 3 and 4). Cells were fixed 24 hours after the start of treatment and stained for collagen-I (red) and nuclei (blue). Merged and individual channel images are shown for each condition. Quantification of collagen-I average fluorescence intensity reveals increased expression upon TGF-β1 treatment, which is partially inhibited by sPIF co-treatment. This finding may suggest a reduced transition of fibroblasts into myofibroblasts, a process typically associated with more fibrotic conditions. Images were acquired at 20× magnification. Results are presented as mean ± SEM after normalization to the total amount of nucleus per image. ^*^p<0.05; ^**^p<0.01; ^***^p<0.001. One-way ANOVA, Tukey post hoc test.

# Supplementary Figure 8. sPIF reduces α-smooth muscle actin (αSMA) expression in human cardiac fibroblasts following TGF-β1 exposure. Human cardiac fibroblasts were pre-treated with sPIF (50 ng/mL) for 1 hour (Columns 2 and 4), followed by the addition of TGF-β1 (20 ng/mL) in the respective conditions (Columns 3 and 4). Cells were fixed 24 hours after the start of treatment and stained for αSMA (red) and nuclei (blue). Merged and individual channel images are shown for each condition. Cells treated with TGF-β1 exhibited a significant increase in αSMA expression compared to the control group, consistent with a myofibroblastic phenotype. Treatment with sPIF attenuated αSMA expression. Images were acquired at 20× magnification. Results are presented as mean ± SEM after normalization to the total amount of nucleus per image. ^*^p<0.05; ^**^p<0.01; ^***^p<0.001. One-way ANOVA, Tukey post hoc test.

# Supplementary Figure 9. Wound healing assay in human cardiac fibroblast monolayers at different time points (0-30 h) after treatment with vehicle, TGF-β1 (20 ng/mL), sPIF (50 ng/mL), or TGF-β1 + sPIF. (A) Healing rate at 1 hour, (B) healing rate at 3 hours, (C) healing rate at 6 hours, (D) healing rate at 24 hours, and (E) healing rate at 30 hours. Quantification of wound closure revealed that TGF-β1 significantly delayed healing at 24 h and 30 h, consistent with fibroblast differentiation into strongly adherent, contractile αSMA-positive myofibroblasts. Co-treatment with sPIF partially reversed this effect, improving wound closure at the same time points. Notably, sPIF alone enhanced wound healing as early as 1 h, suggesting a pro-migratory effect. Data are presented as mean ± SEM of n = 6 independent experiments. ^*^p<0.05, ^**^p<0.01, ^***^p<0.001. One-way ANOVA, Tukey post hoc test.

# **Supplementary Figure 10.** The effects of Synthetic PreImplantation Factor (sPIF) on the proliferation of human breast cancer cell lines. Viability of (A) MCF-7, (B) T47D, and (C) MDA-MB-231 cells treated by sPIF. Viability of irradiated (4 Gy) (D) MCF-7, (E) T47D, and (F) MDA-MB-231 cells treated by sPIF. Data are expressed as mean ± SEM, n=6-10 replicates/group; ^*^p<0.05, ^**^p<0.01 and ^***^p<0.01, one-way ANOVA, Tukey post hoc test.

a)

b)

# Supplementary Figure 12. Full-length, unmodified Ponceau-stained membranes and cropped Western blot images of mothers against decapentaplegic homolog 2/3 (SMAD2/3 52 and 60 kDa) and glycerinaldehyde-3-phosphate dehydrogenase (GAPDH 37 kDa) at week 1. a) Photo of the uncropped and unmodified Ponceau-stained membrane used later for the detection of SMAD2/3 and GAPDH at week 1. The efficiency of protein transfer onto a nitrocellulose membrane was checked using Ponceau staining. Images were captured by the camera of an Apple iPhone 8. b) Photo of horizontally and vertically cut nitrocellulose membrane strips after incubation with primary (SMAD2/3 and GAPDH) and secondary (IRDye 800CW Goat Anti-Rabbit) antibodies, before scanning. C: control treated with vehicle, MW: Molecular weight, NC: negative control, RT: radiotherapy treated with vehicle, RT+sPIF: radiotherapy treated with synthetic preimplantation factor. The black lines on the Western blot indicate where the original nitrocellulose membrane was cut before blocking and/or incubating with the primary antibody. Then, the membrane pieces were assembled and scanned on the Odyssey CLx. Lanes 11, 12, and 14 were used as representative bands in Figure 3c.

a)

b)

# **Supplementary Figure 13.** Full-length, unmodified Ponceau-stained membranes and cropped Western blot images of mothers against decapentaplegic homolog 2/3 (SMAD2/3 52 and 60 kDa) and glycerinaldehyde-3-phosphate dehydrogenase (GAPDH 37 kDa) at week 1. a) Photo of the uncropped and unmodified Ponceau-stained membrane used later for the detection of SMAD2/3 and GAPDH at week 1. The efficiency of protein transfer onto a nitrocellulose membrane was checked using Ponceau staining. Images were captured by the camera of an Apple iPhone 8. b) Photo of horizontally and vertically cut nitrocellulose membrane strips after incubation with primary (SMAD2/3 and GAPDH) and secondary (IRDye 800CW Goat Anti-Rabbit) antibodies, before scanning. C: control treated with vehicle, MW: Molecular weight, NC: negative control, RT: radiotherapy treated with vehicle, RT+sPIF: radiotherapy treated with synthetic preimplantation factor. The black lines on the Western blot indicate where the original nitrocellulose membrane was cut before blocking and/or incubating with the primary antibody. Then, the membrane pieces were assembled and scanned on the Odyssey CLx. Lanes 11, 12, and 14 were used as representative bands in Figure 3c.

a)

b)

# **Supplementary Figure 14.** Full-length, unmodified Ponceau-stained membranes and cropped Western blot images of mothers against decapentaplegic homolog 2/3 (SMAD2/3 52 and 60 kDa) and glycerinaldehyde-3-phosphate dehydrogenase (GAPDH 37 kDa) at week 3. a) Photo of the uncropped and unmodified Ponceau-stained membrane used later for the detection of SMAD2/3 and GAPDH at week 3. The efficiency of protein transfer onto a nitrocellulose membrane was checked using Ponceau staining. Images were captured by the camera of an Apple iPhone 8. b) Photo of horizontally and vertically cut nitrocellulose membrane strips after incubation with primary (SMAD2/3 and GAPDH) and secondary (IRDye 800CW Goat Anti-Rabbit) antibodies, before scanning. C: control treated with vehicle, MW: Molecular weight, NC: negative control, RT: radiotherapy treated with vehicle, RT+sPIF: radiotherapy treated with synthetic preimplantation factor. The black lines on the Western blot indicate where the original nitrocellulose membrane was cut before blocking and/or incubating with the primary antibody. Then, the membrane pieces were assembled and scanned on the Odyssey CLx. Lanes 20, 21, and 23 were used as representative bands in Figure 3c.

a)

b)

# **Supplementary Figure 15.** Full-length, unmodified Ponceau-stained membranes and cropped Western blot images of mothers against decapentaplegic homolog 2/3 (SMAD2/3 52 and 60 kDa) and glycerinaldehyde-3-phosphate dehydrogenase (GAPDH 37 kDa) at week 3. a) Photo of the uncropped and unmodified Ponceau-stained membrane used later for the detection of SMAD2/3 and GAPDH at week 3. The efficiency of protein transfer onto a nitrocellulose membrane was checked using Ponceau staining. Images were captured by the camera of an Apple iPhone 8. b) Photo of horizontally and vertically cut nitrocellulose membrane strips after incubation with primary (SMAD2/3 and GAPDH) and secondary (IRDye 800CW Goat Anti-Rabbit) antibodies, before scanning. C: control treated with vehicle, MW: Molecular weight, NC: negative control, RT: radiotherapy treated with vehicle, RT+sPIF: radiotherapy treated with synthetic preimplantation factor. The black lines on the Western blot indicate where the original nitrocellulose membrane was cut before blocking and/or incubating with the primary antibody. Then, the membrane pieces were assembled and scanned on the Odyssey CLx. Lanes 20, 21, and 23 were used as representative bands in Figure 3c.

a)

b)

**Supplementary Figure 16.** Full-length, unmodified Ponceau-stained membranes and cropped Western blot images of mothers against decapentaplegic homolog 2/3 (SMAD2/3 52 and 60 kDa) and glycerinaldehyde-3-phosphate dehydrogenase (GAPDH 37 kDa) at week 15. a) Photo of the uncropped and unmodified Ponceau-stained membrane used later for the detection of SMAD2/3 and GAPDH at week 15. The efficiency of protein transfer onto a nitrocellulose membrane was checked using Ponceau staining. Images were captured by the camera of an Apple iPhone 8. b) Photo of horizontally and vertically cut nitrocellulose membrane strips after incubation with primary (SMAD2/3 and GAPDH) and secondary (IRDye 800CW Goat Anti-Rabbit) antibodies, before scanning. C: control treated with vehicle, MW: Molecular weight, NC: negative control, RT: radiotherapy treated with vehicle, RT+sPIF: radiotherapy treated with synthetic preimplantation factor. The black lines on the Western blot indicate where the original nitrocellulose membrane was cut before blocking and/or incubating with the primary antibody. Then, the membrane pieces were assembled and scanned on the Odyssey CLx. Lanes 11, 12, and 14 were used as representative bands in Figure 3c.

a)

b)

# **Supplementary Figure 17.** Full-length, unmodified Ponceau-stained membranes and cropped Western blot images of sarcoendoplasmic reticulum calcium ATP-ase 2a isoform (SERCA2a 114 and 140 kDa) and Tubulin (52 kDa) at week 1. a) Photo of the uncropped and unmodified Ponceau-stained membrane used later for the detection of SERCA2a and Tubulin at week 1. The efficiency of protein transfer onto a nitrocellulose membrane was checked using Ponceau staining. Images were captured by the camera of an Apple iPhone 8. b) Photo of horizontally and vertically cut nitrocellulose membrane strips after incubation with primary (SERCA2a and Tubulin) and secondary (IRDye 800CW Goat Anti-Rabbit) antibodies, before scanning. C: control treated with vehicle, MW: Molecular weight, NC: negative control, RT: radiotherapy treated with vehicle, RT+sPIF: radiotherapy treated with synthetic preimplantation factor. The black lines on the Western blot indicate where the original nitrocellulose membrane was cut before blocking and/or incubating with the primary antibody. Then, the membrane pieces were assembled and scanned on the Odyssey CLx. Lanes 3, 4, and 6 were used as representative bands in Figure 3c.

a)

b)

# **Supplementary Figure 18.** Full-length, unmodified Ponceau-stained membranes and cropped Western blot images of sarcoendoplasmic reticulum calcium ATP-ase 2a isoform (SERCA2a 114 and 140 kDa) and Tubulin (52 kDa) at week 1. a) Photo of the uncropped and unmodified Ponceau-stained membrane used later for the detection of SERCA2a and Tubulin at week 1. The efficiency of protein transfer onto a nitrocellulose membrane was checked using Ponceau staining. Images were captured by the camera of an Apple iPhone 8. b) Photo of horizontally and vertically cut nitrocellulose membrane strips after incubation with primary (SERCA2a and Tubulin) and secondary (IRDye 800CW Goat Anti-Rabbit) antibodies, before scanning. C: C: control treated with vehicle, MW: Molecular weight, NC: negative control, RT: radiotherapy treated with vehicle, RT+sPIF: radiotherapy treated with synthetic preimplantation factor. The black lines on the Western blot indicate where the original nitrocellulose membrane was cut before blocking and/or incubating with the primary antibody. Then, the membrane pieces were assembled and scanned on the Odyssey CLx. Lanes 3, 4, and 6 were used as representative bands in Figure 7f.

a)

b)

# **Supplementary Figure 19.** Full-length, unmodified Ponceau-stained membranes and cropped Western blot images of sarcoendoplasmic reticulum calcium ATP-ase 2a isoform (SERCA2a 114 and 140 kDa) and Tubulin (52 kDa) at week 3. a) Photo of the uncropped and unmodified Ponceau-stained membrane used later for the detection of SERCA2a and Tubulin at week 3. The efficiency of protein transfer onto a nitrocellulose membrane was checked using Ponceau staining. Images were captured by the camera of an Apple iPhone 8. b) Photo of horizontally and vertically cut nitrocellulose membrane strips after incubation with primary (SERCA2a and Tubulin) and secondary (IRDye 800CW Goat Anti-Rabbit) antibodies, before scanning. C: control treated with vehicle, MW: Molecular weight, NC: negative control, RT: radiotherapy treated with vehicle, RT+sPIF: radiotherapy treated with synthetic preimplantation factor. The black lines on the Western blot indicate where the original nitrocellulose membrane was cut before blocking and/or incubating with the primary antibody. Then, the membrane pieces were assembled and scanned on the Odyssey CLx. Lanes 7, 8, and 10 were used as representative bands in Figure 7f.

a)

b)

# **Supplementary Figure 20.** Full-length, unmodified Ponceau-stained membranes and cropped Western blot images of sarcoendoplasmic reticulum calcium ATP-ase 2a isoform (SERCA2a 114 and 140 kDa) and Tubulin (52 kDa) at week 3. a) Photo of the uncropped and unmodified Ponceau-stained membrane used later for the detection of SERCA2a and Tubulin at week 3. The efficiency of protein transfer onto a nitrocellulose membrane was checked using Ponceau staining. Images were captured by the camera of an Apple iPhone 8. b) Photo of horizontally and vertically cut nitrocellulose membrane strips after incubation with primary (SERCA2a and Tubulin) and secondary (IRDye 800CW Goat Anti-Rabbit) antibodies, before scanning. C: C: control treated with vehicle, MW: Molecular weight, NC: negative control, RT: radiotherapy treated with vehicle, RT+sPIF: radiotherapy treated with synthetic preimplantation factor. The black lines on the Western blot indicate where the original nitrocellulose membrane was cut before blocking and/or incubating with the primary antibody. Then, the membrane pieces were assembled and scanned on the Odyssey CLx. Lanes 7, 8, and 10 were used as representative bands in Figure 7f.

a)

b)

# **Supplementary Figure 21.** Full-length, unmodified Ponceau-stained membranes and cropped Western blot images of sarcoendoplasmic reticulum calcium ATP-ase 2a isoform (SERCA2a 114 and 140 kDa) and Tubulin (52 kDa) at week 15. a) Photo of the uncropped and unmodified Ponceau-stained membrane used later for the detection of SERCA2a and Tubulin at week 3. The efficiency of protein transfer onto a nitrocellulose membrane was checked using Ponceau staining. Images were captured by the camera of an Apple iPhone 8. b)Photo of horizontally and vertically cut nitrocellulose membrane strips after incubation with primary (SERCA2a and Tubulin) and secondary (IRDye 800CW Goat Anti-Rabbit) antibodies, before scanning. C: C: control treated with vehicle, MW: Molecular weight, NC: negative control, RT: radiotherapy treated with vehicle, RT+sPIF: radiotherapy treated with synthetic preimplantation factor. The black lines on the Western blot indicate where the original nitrocellulose membrane was cut before blocking and/or incubating with the primary antibody. Then, the membrane pieces were assembled and scanned on the Odyssey CLx. Lanes 27, 28, and 30 were used as representative bands in Figure 7f

a)

b)

**Supplementary Figure 22.** Full-length, unmodified Ponceau-stained membranes and cropped Western blot images of BCL2-associated X apoptosis regulator gene (BAX) at week 1. a) Photo of the uncropped and unmodified Ponceau-stained membrane used later for the detection of BAX and GAPDH at week 1. The efficiency of protein transfer onto a nitrocellulose membrane was checked using Ponceau staining. Images were captured by the camera of an Apple iPhone 8. b) Photo of horizontally and vertically cut nitrocellulose membrane strips after incubation with primary (BAX) and secondary (IRDye 800CW Goat Anti-Rabbit) antibodies, before scanning. C: control treated with vehicle, MW: Molecular weight, NC: negative control, RT: radiotherapy treated with vehicle, RT+sPIF: radiotherapy treated with synthetic preimplantation factor. The black lines on the Western blot indicate where the original nitrocellulose membrane was cut before blocking and/or incubating with the primary antibody. Then, the membrane pieces were assembled and scanned on the Odyssey CLx. Lanes 9, 10, and 11 were used as representative bands in Figure 8d.

a)

b)

**Supplementary Figure 23.** Full-length, unmodified Ponceau-stained membranes and cropped Western blot images of glycerinaldehyde-3-phosphate dehydrogenase (GAPDH 37 kDa) at week 1. a) Photo of the uncropped and unmodified Ponceau-stained membrane used later for the detection of BAX and GAPDH at week 1. The efficiency of protein transfer onto a nitrocellulose membrane was checked using Ponceau staining. Images were captured by the camera of an Apple iPhone 8. b) Photo of horizontally and vertically cut nitrocellulose membrane strips after incubation with primary (GAPDH) and secondary (IRDye 800CW Goat Anti-Rabbit) antibodies, before scanning. C: control treated with vehicle, MW: Molecular weight, NC: negative control, RT: radiotherapy treated with vehicle, RT+sPIF: radiotherapy treated with synthetic preimplantation factor. The black lines on the Western blot indicate where the original nitrocellulose membrane was cut before blocking and/or incubating with the primary antibody. Then, the membrane pieces were assembled and scanned on the Odyssey CLx. Lanes 9, 10, and 11 were used as representative bands in Figure 8d.

a)

b)

**Supplementary Figure 24.** Full-length, unmodified Ponceau-stained membranes and cropped Western blot images of BCL2-associated X apoptosis regulator gene (BAX) at week 3. a) Photo of the uncropped and unmodified Ponceau-stained membrane used later for the detection of BAX and GAPDH at week 3. The efficiency of protein transfer onto a nitrocellulose membrane was checked using Ponceau staining. Images were captured by the camera of an Apple iPhone 8. b) Photo of horizontally and vertically cut nitrocellulose membrane strips after incubation with primary (BAX) and secondary (IRDye 800CW Goat Anti-Rabbit) antibodies, before scanning. C: control treated with vehicle, MW: Molecular weight, NC: negative control, RT: radiotherapy treated with vehicle, RT+sPIF: radiotherapy treated with synthetic preimplantation factor. The black lines on the Western blot indicate where the original nitrocellulose membrane was cut before blocking and/or incubating with the primary antibody. Then, the membrane pieces were assembled and scanned on the Odyssey CLx. Lanes 19, 20, and 21 were used as representative bands in Figure 8d.

a)

b)

**Supplementary Figure 25.** Full-length, unmodified Ponceau-stained membranes and cropped Western blot images of glycerinaldehyde-3-phosphate dehydrogenase (GAPDH 37 kDa) at week 3. a) Photo of the uncropped and unmodified Ponceau-stained membrane used later for the detection of BAX and GAPDH at week 3. The efficiency of protein transfer onto a nitrocellulose membrane was checked using Ponceau staining. Images were captured by the camera of an Apple iPhone 8. b) Photo of horizontally and vertically cut nitrocellulose membrane strips after incubation with primary (GAPDH) and secondary (IRDye 800CW Goat Anti-Rabbit) antibodies, before scanning. C: control treated with vehicle, MW: Molecular weight, NC: negative control, RT: radiotherapy treated with vehicle, RT+sPIF: radiotherapy treated with synthetic preimplantation factor. The black lines on the Western blot indicate where the original nitrocellulose membrane was cut before blocking and/or incubating with the primary antibody. Then, the membrane pieces were assembled and scanned on the Odyssey CLx. Lanes 19, 20, and 21 were used as representative bands in Figure 8d.

a)

b)

**Supplementary Figure 26.** Full-length, unmodified Ponceau-stained membranes and cropped Western blot images of the BCL2-associated X apoptosis regulator gene (BAX) at week 15. a) Photo of the uncropped and unmodified Ponceau-stained membrane used later for the detection of BAX and GAPDH at week 15. The efficiency of protein transfer onto a nitrocellulose membrane was checked using Ponceau staining. Images were captured by the camera of an Apple iPhone 8. b) Photo of horizontally and vertically cut nitrocellulose membrane strips after incubation with primary (BAX) and secondary (IRDye 800CW Goat Anti-Rabbit) antibodies, before scanning. C: control treated with vehicle, MW: Molecular weight, NC: negative control, RT: radiotherapy treated with vehicle, RT+sPIF: radiotherapy treated with synthetic preimplantation factor. The black lines on the Western blot indicate where the original nitrocellulose membrane was cut before blocking and/or incubating with the primary antibody. Then, the membrane pieces were assembled and scanned on the Odyssey CLx. Lanes 19, 20, and 21 were used as representative bands in Figure 8d.

a)

b)

**Supplementary Figure 27.** Full-length, unmodified Ponceau-stained membranes and cropped Unmodified Western blot images of glycerinaldehyde-3-phosphate dehydrogenase (GAPDH 37 kDa) at week 15. a) Photo of the uncropped and unmodified Ponceau-stained membrane used later for the detection of BAX and GAPDH at week 15. The efficiency of protein transfer onto a nitrocellulose membrane was checked using Ponceau staining. Images were captured by the camera of an Apple iPhone 8. b) Photo of horizontally and vertically cut nitrocellulose membrane strips after incubation with primary (GAPDH) and secondary (IRDye 800CW Goat Anti-Rabbit) antibodies, before scanning. C: control treated with vehicle, MW: Molecular weight, NC: negative control, RT: radiotherapy treated with vehicle, RT+sPIF: radiotherapy treated with synthetic preimplantation factor. The black lines on the Western blot indicate where the original nitrocellulose membrane was cut before blocking and/or incubating with the primary antibody. Then, the membrane pieces were assembled and scanned on the Odyssey CLx. Lanes 19, 20, and 21 were used as representative bands in Figure 8d.

a)

b)

**Supplementary Figure 28.** Full-length, unmodified Ponceau-stained membranes and cropped Western blot images of apoptosis-inducing factor (AIF 67 kDa), Caspase 7 (CASP7 20 and 35 kDa), and Tubulin (52 kDa) at week 1. a) Photo of the uncropped and unmodified Ponceau-stained membrane used later for the detection of AIF, CASP7, BCL2, and Tubulin at week 1. The efficiency of protein transfer onto a nitrocellulose membrane was checked using Ponceau staining. Images were captured by the camera of an Apple iPhone 8. b) Photo of horizontally and vertically cut nitrocellulose membrane strips after incubation with primary (AIF, CASP7, and Tubulin, respectively) and secondary (IRDye 800CW Goat Anti-Rabbit) antibodies, before scanning. C: control treated with vehicle, MW: Molecular weight, NC: negative control, RT: radiotherapy treated with vehicle, RT+sPIF: radiotherapy treated with synthetic preimplantation factor. The black lines on the Western blot indicate where the original nitrocellulose membrane was cut before blocking and/or incubating with the primary antibody. Then, the membrane pieces were assembled and scanned on the Odyssey CLx. Lanes 9, 10, and 11 were used as representative bands in Supplementary Figures 6e and 6f, and Figure 8e.

a)

b)

**Supplementary Figure 29.** Full-length, unmodified Ponceau-stained membranes and cropped Western blot images of caspase 7 (CASP7) and B-cell lymphoma-2 (BCL2 26 kDa) at week 1. a) Photo of the uncropped and unmodified Ponceau-stained membrane used later for the detection of AIF, CASP7, BCL2, and Tubulin at week 1. The efficiency of protein transfer onto a nitrocellulose membrane was checked using Ponceau staining. Images were captured by the camera of an Apple iPhone 8. b) Photo of horizontally and vertically cut nitrocellulose membrane strips after incubation with primary (BCL2 and CASP7, respectively) and secondary (IRDye 800CW Goat Anti-Rabbit) antibodies, before scanning. C: control treated with vehicle, MW: Molecular weight, NC: negative control, RT: radiotherapy treated with vehicle, RT+sPIF: radiotherapy treated with synthetic preimplantation factor. The black lines on the Western blot indicate where the original nitrocellulose membrane was cut before blocking and/or incubating with the primary antibody. Then, the membrane pieces were assembled and scanned on the Odyssey CLx. Lanes 9, 10, and 11 were used as representative bands in Figures 8d and Supplementary Figure 6e.

a)

b)

**Supplementary Figure 30** Full-length, unmodified Ponceau-stained membranes and cropped Western blot images of apoptosis-inducing factor (AIF 67 kDa), Caspase 7 (CASP7 20 and 35 kDa), and Tubulin (52 kDa) at week 3. a) Photo of the uncropped and unmodified Ponceau-stained membrane used later for the detection of AIF, CASP7, BCL2, and Tubulin at week 3. The efficiency of protein transfer onto a nitrocellulose membrane was checked using Ponceau staining. Images were captured by the camera of an Apple iPhone 8. b) Photo of horizontally and vertically cut nitrocellulose membrane strips after incubation with primary (AIF, CASP7 and Tubulin, respectively) and secondary (IRDye 800CW Goat Anti-Rabbit) antibodies, before scanning. C: control treated with vehicle, MW: Molecular weight, NC: negative control, RT: radiotherapy treated with vehicle, RT+sPIF: radiotherapy treated with synthetic preimplantation factor. The black lines on the Western blot indicate where the original nitrocellulose membrane was cut before blocking and/or incubating with the primary antibody. Then, the membrane pieces were assembled and scanned on the Odyssey CLx. Lanes 19, 20, and 21 were used as representative bands in Supplementary Figures 6e and 6f, and Figure 8e.

a)

b)

**Supplementary Figure 31** Full-length, unmodified Ponceau-stained membranes and cropped Western blot images: caspase 7 (CASP7) and B-cell lymphoma-2 (BCL2 26 kDa) at week 3. a) Photo of the uncropped and unmodified Ponceau-stained membrane used later for the detection of AIF, CASP7, BCL2, and Tubulin at week 3. The efficiency of protein transfer onto a nitrocellulose membrane was checked using Ponceau staining. Images were captured by the camera of an Apple iPhone 8. b) Photo of horizontally and vertically cut nitrocellulose membrane strips after incubation with primary (BCL2 and CASP7, respectively) and secondary (IRDye 800CW Goat Anti-Rabbit) antibodies, before scanning. C: control treated with vehicle, MW: Molecular weight, NC: negative control, RT: radiotherapy treated with vehicle, RT+sPIF: radiotherapy treated with synthetic preimplantation factor. The black lines on the Western blot indicate where the original nitrocellulose membrane was cut before blocking and/or incubating with the primary antibody. Then, the membrane pieces were assembled and scanned on the Odyssey CLx. Lanes 19, 20, and 21 were used as representative bands in Figures 8d and Supplementary Figure 6e.

a)

b)

**Supplementary Figure 32.** Full-length, unmodified Ponceau-stained membranes and cropped Western blot images of apoptosis-inducing factor (AIF 67 kDa), Caspase 7 (CASP7 20 and 35 kDa), B-cell lymphoma-2 (BCL2 26 kDa) and Tubulin (52 kDa) at week 15. a) Photo of the uncropped and unmodified Ponceau-stained membrane used later for the detection of AIF, CASP7, BCL2, and Tubulin at week 15. The efficiency of protein transfer onto a nitrocellulose membrane was checked using Ponceau staining. Images were captured by the camera of an Apple iPhone 8. b) Photo of horizontally and vertically cut nitrocellulose membrane strips after incubation with primary (AIF, CASP7, BCL2, and Tubulin, respectively) and secondary (IRDye 800CW Goat Anti-Rabbit) antibodies, before scanning. C: control treated with vehicle, MW: Molecular weight, NC: negative control, RT: radiotherapy treated with vehicle, RT+sPIF: radiotherapy treated with synthetic preimplantation factor. The black lines on the Western blot indicate where the original nitrocellulose membrane was cut before blocking and/or incubating with the primary antibody. Then, the membrane pieces were assembled and scanned on the Odyssey CLx. Lanes 16, 17, and 18 were used as representative bands in Supplementary Figures 6e and 6f, and Figures 8d and 8e.

# **REFERENCES**

1.	Sárközy M, Gáspár R, Zvara Á, Kiscsatári L, Varga Z, Kővári B, et al. Selective Heart Irradiation Induces Cardiac Overexpression of the Pro-hypertrophic miR-212. *Frontiers in oncology* (2019) **9**:598. doi:10.3389/fonc.2019.00598.

2.	Kovács MG, Kovács ZZ, Varga Z, Szűcs G, Freiwan M, Farkas K, et al. Investigation of the Antihypertrophic and Antifibrotic Effects of Losartan in a Rat Model of Radiation-Induced Heart Disease. *International journal of molecular sciences* (2021) **22**. doi:10.3390/ijms222312963.

3.	Sárközy M, Watzinger S, Kovács ZZ, Acar E, Márványkövi F, Szűcs G, et al. Neuregulin-1β Improves Uremic Cardiomyopathy and Renal Dysfunction in Rats. *JACC. Basic to translational science* (2023) **8**:1160–76. doi:10.1016/j.jacbts.2023.03.003.

4.	Freiwan M, Kovács MG, Kovács ZZ, Szűcs G, Dinh H, Losonczi R, et al. Investigation of the Antiremodeling Effects of Losartan, Mirabegron and Their Combination on the Development of Doxorubicin-Induced Chronic Cardiotoxicity in a Rat Model. *International journal of molecular sciences* (2022) **23**. doi:10.3390/ijms23042201.

5.	Galla Z, Rácz G, Grecsó N, Baráth Á, Kósa M, Bereczki C, et al. Improved LC-MS/MS method for the determination of 42 neurologically and metabolically important molecules in urine. *Journal of chromatography. B, Analytical technologies in the biomedical and life sciences* (2021) **1179**:122846. doi:10.1016/j.jchromb.2021.122846.

6.	Polyák H, Galla Z, Nánási N, Cseh EK, Rajda C, Veres G, et al. The Tryptophan-Kynurenine Metabolic System Is Suppressed in Cuprizone-Induced Model of Demyelination Simulating Progressive Multiple Sclerosis. *Biomedicines* (2023) **11**. doi:10.3390/biomedicines11030945.

7.	Taylor SC, Nadeau K, Abbasi M, Lachance C, Nguyen M, Fenrich J. The Ultimate qPCR Experiment: Producing Publication Quality, Reproducible Data the First Time. *Trends in biotechnology* (2019) **37**:761–74. doi:10.1016/j.tibtech.2018.12.002.

8.	Kovács ZZ, Szűcs G, Freiwan M, Kovács MG, Márványkövi FM, Dinh H, et al. Comparison of the antiremodeling effects of losartan and mirabegron in a rat model of uremic cardiomyopathy. *Scientific reports* (2021) **11**:17495. doi:10.1038/s41598-021-96815-5.

9.	Molnár B, Kinyua NI, Mótyán G, Leits P, Zupkó I, Minorics R, et al. Regioselective synthesis, physicochemical properties and anticancer activity of 2-aminomethylated estrone derivatives. *The Journal of steroid biochemistry and molecular biology* (2022) **219**:106064. doi:10.1016/j.jsbmb.2022.106064.
